# Click chemistry-enabled gold nanorods for sensitive detection and viability evaluation of copper(II)-reducing bacteria

**DOI:** 10.1016/j.mtbio.2025.101453

**Published:** 2025-01-04

**Authors:** Tongtong Tian, Wenjing Yang, Xiaohuan Wang, Te Liu, Baishen Pan, Wei Guo, Beili Wang

**Affiliations:** a Department of Laboratory Medicine, Zhongshan Hospital, Fudan University, 136 Yi Xue Yuan Road, Shanghai, 200032, PR China; b Department of Laboratory Medicine, Shanghai Geriatric Medical Center, Shanghai, 201100, PR China; c Department of Laboratory Medicine, Wusong Central Hospital, Baoshan District, Shanghai, 200940, PR China; d Department of Laboratory Medicine, Xiamen Branch, Zhongshan Hospital, Fudan University, 361015, PR China; e Shanghai Geriatric Institute of Chinese Medicine, Shanghai University of Traditional Chinese Medicine, No. 725 South Wan Ping Road, Shanghai, 200031, PR China

**Keywords:** Gold nanorods, Click chemistry, Microbial sensor, Biomarker, POCT

## Abstract

The rise of antibiotic resistance poses a significant and ongoing challenge to public health, with pathogenic bacteria remaining a persistent threat. Traditional culture methods, while considered the gold standard for bacterial detection and viability assessment, are time-consuming and labor-intensive. To address this limitation, we developed a novel point-of-care (POC) detection method leveraging citrate- and alkyne-modified gold nanorods (AuNRs) synthesized with click chemistry properties. These AuNRs exhibit superior biocompatibility and enhanced quantitative performance compared to conventional surfactant-modified AuNRs. Our method, termed AuNRs–bacteria-initiated click chemistry (AuNRs–BICC), detects Cu^II^-reducing bacteria by quantifying AuNRs bound to a biosensing interface via bacteria-mediated Cu^II^ reduction to Cu^I^ and subsequent click chemistry with biosensing interface of azide modifications. Using dark-field microscopy (DFM), we demonstrated a strong linear correlation between AuNR counts and the logarithm of bacterial concentration for both Gram-negative *Escherichia coli* (including KPC-2-expressing antibiotic-resistant strains) and Gram-positive *Staphylococcus aureus* across a range of 10^1^ to 10^7^ cells, achieving a remarkable detection limit of 10^1^ cells. The AuNRs–BICC biosensor exhibits high selectivity for target bacterial strains and provides rapid detection within 3 h. Furthermore, it can assess bacterial viability in the presence of various antibiotics, including meropenem, ceftriaxone and tetracycline, suggesting its potential for rapid antibiotic susceptibility testing and facilitating timely clinical intervention for infectious diseases.

## Highlights

• A POC microbial sensor via single-particles enumeration under dark-field microscopy was developed.
• This sensor can be utilized for bacterial quantification, viability assessment and antimicrobial susceptibility testing.
• Compared to traditional methods, this sensor demonstrates great potential for clinical POC diagnosis of bacterial infections.

## Graphical abstract

**Figure:**
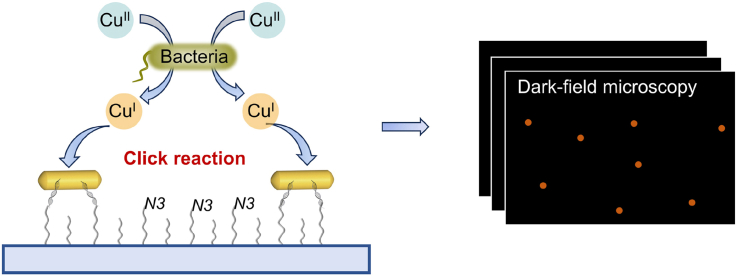

## 1 Introduction

Pathogenic bacteria represent a significant threat to public health, causing a wide range of human diseases. The relatively low infectious dose required by many bacterial species, coupled with the alarming rise of antimicrobial resistance (estimated to have contributed to 4.95 million deaths in 2019 [[1], [2], [3]]), exacerbates this challenge. Common bacterial infections encompass foodborne illnesses, urinary tract infections, sexually transmitted infections, and healthcare-associated infections [4,5]. The substantial economic burden associated with these infections, along with the widespread prevalence of antibiotic resistance, underscores the urgent need for rapid and reliable point-of-care diagnostic assays for bacterial identification and antimicrobial susceptibility testing (AST).

Traditional culture-based methods for bacterial detection, while considered the gold standard, are time-consuming and labor-intensive. This has spurred the development of alternative strategies, including polymerase chain reaction (PCR), enzyme-linked immunosorbent assay (ELISA), and matrix-assisted laser desorption/ionization time-of-flight (MALDI-TOF) mass spectrometry (advantages, disadvantages, and applications summarized in Table S1) [[6], [7], [8], [9]]. However, these methods often require specialized equipment and trained personnel, hindering their widespread implementation, particularly in resource-limited settings [[10], [11], [12], [13]]. Recent research has highlighted bacterial adaptation mechanisms to copper-rich environments, involving copper-binding systems and reductases [[14], [15], [16], [17], [18], [19], [20], [21]]. This copper-related pathway has inspired the development of novel biosensing strategies. Click chemistry, particularly the copper-catalyzed azide-alkyne cycloaddition (CuAAC) reaction [22,23], offers highly efficient and selective chemical reactions with minimal byproducts, ideal for biological applications. Building upon this, colorimetric assays employing bacteria-instructed click chemistry with gold nanoparticles (AuNPs) and electrochemically mediated atom transfer radical polymerization have shown promise for bacterial detection [14,[24], [25], [26]]. Nevertheless, these assays suffer from limitations, including susceptibility to environmental interference, low sensitivity, and the need for calibration, which hinder accurate quantification and drug resistance evaluation.

Gold nanorods (AuNRs) are promising nanomaterials for various applications due to their unique optical properties (plasmonics), tunable resonance frequency dependent on their aspect ratio, and facile synthesis [[27], [28], [29]]. However, traditional surfactant-modified AuNRs suffer from cytotoxicity and poor biocompatibility, limiting their use in biological systems [27]. Recent advances in citrate-capped AuNRs have addressed these limitations, paving the way for their use in a variety of bio-applications [30,31]. Single-particle enumeration using DFM has emerged as a powerful technique for analyzing metallic nanocrystals, such as gold nanoparticles (AuNPs), offering high sensitivity and the ability to analyze individual particles based on their strong plasmon scattering [32]. To our knowledge, the application of citrate-capped AuNRs and DFM imaging for sensitive, portable, and cost-effective bacterial quantification and drug resistance evaluation using copper-catalyzed azide-alkyne cycloaddition (CuAAC) remains unexplored.

Here, we present a novel point-of-care (POC) bacterial detection method, termed AuNRs–BICC, employing newly synthesized citrate- and alkyne-modified AuNRs with enhanced scattering properties, click chemistry functionality, and excellent biocompatibility. This method leverages AuNR enumeration and DFM imaging. To mitigate non-specific binding on the coverslip surface, we optimized surface treatment using a PLL–PEG–azide polymer, effectively minimizing background signals and non-specific physisorption, consistent with previous reports [[33], [34], [35], [36], [37], [38]]. To ensure assay stability, reactions were performed at a constant temperature to prevent nanoparticle aggregation or dispersion, and pH was controlled using a buffer solution to maintain surface charge stability. The AuNRs–BICC assay relies on the bacterial reduction of exogenous Cu^II^ to Cu^I^, which subsequently catalyzes the CuAAC reaction, leading to AuNR immobilization on the coverslip surface and enabling bacterial quantification via DFM (illustrated in Fig. 2). This AuNRs–BICC sensor demonstrates high selectivity for specific bacterial strains, enabling their detection in complex samples. Furthermore, the platform's adaptability extends to antibiotic susceptibility testing.

## 2 Material and methods

### 2.1 Materials

Tetrachloroauric(III) acid tetrahydrate (HAuCl_4_⋅4H_2_O), hexadecyltrimethylammonium bromide (CTAB), hydroquinone, sodium borohydride (NaBH_4_), potassium hydroxide (KOH) and silver nitrate (AgNO_3_) were purchased from Sinopharm Chemical Reagent Co, Ltd. (Shanghai, China). Meropenem was purchased from Shanghai Yuanye Biotechnology Co., Ltd. (Shanghai, China). Levofloxacin was from Shanghai Macklin Biochemical Co., Ltd. (Shanghai, China). Ceftriaxone disodium salt hemiheptahydrate and tetracycline hydrochloride were from J&K Scientific (Shanghai, China). N-(3-(Dimethylamino)propyl-N′-ethylcarbodiimide)hydrochloride (EDC), N-hydroxysulfosuccinimide sodium salt (NHS), Tris-(2-carboxyethyl)-phosphine hydrochloride (TCEP), copper(II) chloride (CuCl_2_), DNase I (Deoxyribonuclease I), Sodium polystyrenesulfonate (Na-PSS, Mw = 70 kDa) and sodium ascorbate were purchased from Sigma-Aldrich (Shanghai, China). Poly(L-lysine)-poly(ethyleneglycol)-azide (PLL-PEG-azide, PLL(3 kDa)-PEG(5 kDa)-azide) and Poly(L-lysine)-poly(ethyleneglycol) (PLL-PEG, PLL(3 kDa)-PEG(4.6 kDa)) were obtained from NanosoftPolymers (NorthCarolina, USA). Carboxyl-modified magnetic beads (COOH-MBs, 10 mg/mL) were purchased from Invitrogen (California, USA). Streptavidin-modified magnetic beads (SA-MBs, 10 mg/mL) were purchased from MedChemExpress (New Jersey, USA). Luria-Bertani broth medium (LB) and Tryptone Soy Agar (TSA) were from Beijing Land Bridge Technology Co., Ltd. (Beijing, China). All chemicals and reagents were analytical grade or higher, and used as received without further purification. *Escherichia coli* (*E. coli*, ATCC 25922) and *Staphylococcus aureus* (*S. aureus*, ATCC 25923) were obtained from Bioyong Technologics Inc (Beijing, China). All oligonucleotides (the sequences of oligonucleotides were described in Table S2) were synthesized by Sangon Biotech (Shanghai, China). The Flexdym film (thickness of 2 mm) was procured from Eden Tech Featured Inc. (Paris, France). Deionized (DI) water (Millipore Milli-Q grade, 18.2 MΩ) was used in all the experiments.

### 2.2 Apparatus

The UV–vis absorption spectrum of the colloidal AuNR solution was acquired using an Agilent HP8453 UV–vis spectrophotometer. AuNR morphology and dimensions were characterized using a JEOL JEM-2011 transmission electron microscope (TEM) operating at 200 kV. Zeta (ζ) potential and AuNR size measurements were conducted using a Malvern Zetasizer Nano ZS (ZS90-2027). All dark-field images were acquired using a 100 × Leica dark-field condenser coupled to a dark-field microspectroscopy imaging system equipped with a true-color CCD camera.

### 2.3 Synthesis of the citrate-capped AuNRs

Citrate-capped AuNRs were synthesized using a seed-mediated method [39] followed by a poly(4-styrenesulfonic acid) (PSS)-mediated ligand exchange [40]. Briefly, cetyltrimethylammonium bromide (CTAB)-capped AuNRs were synthesized and purified via three centrifugation and redispersion (C/R) cycles in 0.15 wt% Na-PSS to remove CTAB. Subsequently, the resulting PSS-coated AuNRs underwent two additional C/R cycles in 2 mM sodium citrate to replace PSS with citrate, yielding stable citrate-capped AuNR sols.

### 2.4 Preparation of sample chambers and azide functionalization of the coverslip surface (azide–functionalized coverslip)

Sterile, enclosed sample chambers were fabricated by bonding a Flexdym coverslip containing 5 mm diameter holes to a glass coverslip. The coverslip surface was then modified with PEG using a method adapted from a previous report [41]. Briefly, the sample chambers were etched with vacuum plasma for 10 min (PDC-002, Harrick Plasma Inc., USA). A 30 μL mixture of PLL-PEG and PLL-PEG-azide (1 mg/mL each, 1:1 ratio) was then introduced into each chamber and incubated for 1 h. Following incubation, the modified chambers were washed with PBS (20 mM phosphate, 120 mM NaCl, pH 7.4) to remove excess PEG. Finally, a second Flexdym coverslip with a single injection micro-hole was placed onto the modified coverslip, creating a sealed and sterile reactor.

### 2.5 Formation of alkyne-modified dsDNA assembly on AuNRs

AuNRs were functionalized with alkyne-modified thiolated DNA (alkyne–DNA–SH) via Au–S bond formation. First, alkyne–DNA–SH (3 μL, 100 μM) was reduced with TCEP (3 μL, 5 mM) in 100 mM Tris-HCl (pH 7.0) at 25 °C for 1 h. This solution was then added to 1 mL of AuNR solution (1 mg/mL) and incubated at 4 °C for 24 h. Excess reagents and unbound DNA were removed by centrifugation at 7000 rpm for 10 min, followed by resuspension of the AuNR pellet in 1 mL of deionized (DI) water. To form the double-stranded DNA (dsDNA)–AuNR conjugate, complementary DNA (cDNA) (100 μM) was added to the alkyne–DNA–functionalized AuNRs. The mixture was heated to 95 °C for 10 min and then slowly cooled to room temperature (25 °C). Finally, unreacted cDNA was removed by centrifugation at 7000 rpm for 10 min.

### *2.6* *Bacteria-instructed click chemistry between the azide–functionalized coverslip and alkyne*–*modified AuNRs*

The bacterial detection assay was initiated by mixing CuCl_2_ (10 μL, 10 μM) with varying concentrations of bacteria (10 μL, ranging from 0 to 10^8^ cells in total) and incubating for 10 min. This allowed for bacterial reduction of Cu^II^ to Cu^I^. Next, alkyne-modified AuNRs (10 μL, 1 mg/mL) were added to the mixture, and the combined solution was introduced onto azide-functionalized coverslips. The reaction was allowed to proceed at 37 °C for 2 h. The bacteria-initiated click chemistry reaction was then quenched by removing the reaction mixture, followed by three washes with PBS. Finally, the number of immobilized AuNRs was quantified using DFM.

### 2.7 Bacterial capture and isolation using aptamer-based magnetic separation

The synthesis of streptavidin-modified magnetic beads conjugated with biotinylated aptamers (SA–MBs–aptamers) and carboxyl-modified magnetic beads conjugated with amine-modified aptamers (COOH–MBs–aptamers) is described in the Supplementary Material. Briefly, 1 mL of bacterial sample (containing *E. coli* or *S. aureus*) was incubated with 100 μL of either COOH–MBs–aptamers or SA–MBs–aptamers at 25 °C for 2 h with gentle agitation. Magnetic separation (0.3 T for 10 s) was then used to isolate the bead-bound bacteria. After washing with deionized water to remove non-specifically bound bacteria, DNase I (1 unit/μL) was added to release the magnetically enriched bacteria from the aptamers. Following a 10-min incubation, the released bacteria were used for subsequent detection via azide-functionalized coverslip-mediated click chemistry with alkyne-modified gold nanorods (AuNRs).

### 2.8 Detection of viable bacteria from human plasma

Human plasma from a healthy volunteer was spiked with *E. coli* to achieve final concentrations of approximately 0, 250, 500, 1000, and 2000 cells per 30 μL. Streptavidin-modified magnetic beads conjugated with biotinylated aptamers (SA–MBs–aptamers) were then added to capture and magnetically separate the *E. coli*. DNase I treatment released the enriched bacteria, which were subsequently quantified using the AuNRs-based click chemistry method in the presence of Cu^II^.

### 2.9 Bacteria-instructed single nanoparticle click assay for drug resistance

Antibiotic susceptibility testing (AST) was performed by incubating *E. coli* (10^8^ cells) with meropenem, ceftriaxone, tetracycline, and levofloxacin at concentrations of 0.01, 0.1, and 1 mg/mL for 0, 1, 2, 4, 6, and 8 h. Following incubation, bacterial cells were harvested by centrifugation (7000 g, 3 min) and washed twice with deionized water. The resulting *E. coli* were then quantified using both a bacteria-instructed single-nanoparticle click assay and a standard bacterial culture method.

### 2.10 Analysis of drug sensitivity of different bacteria

*E. coli* ATCC 25922 and *E. coli* expressing KPC-2 (5000 cells each) were co-incubated with meropenem, ceftriaxone, tetracycline, and levofloxacin (0.01, 0.1, and 1 mg/mL) for 90 min. Antibiotic susceptibility was then determined using both AuNRs-BICC and standard culture methods.

### 2.11 Utilizing dark-field microscopic imaging and analyzing data

Image acquisition: all dark-field images were captured using a Leica DMi8C/HRS-300 dark-field microspectroscopy imaging system equipped with a true-color CCD sensor. Consistent imaging conditions were maintained for all reaction systems throughout the experiments. Images of ultrapure water were used as a reference background. Image selection and analysis: eight images were acquired for each sample, taken from distinct positions near the center of the reaction well. Data analysis was performed using ImageJ software (version 1.45). A central area measuring 300 × 300 μm within each image was selected for counting AuNRs. The 'analyze particles' function was used to count AuNRs within a particle size range of 2–12 pixels. Quantification: the number of AuNRs was calculated using Equation (1).:

(1) $$N_{\text{net}}\;{=N}_{\text{bacteria}\;}{‐\;N}_{\text{negative}}$$

where $N_{\text{net}}$ represents the net counts of AuNRs, $N_{\text{negative}}$ signifies the adjusted count of AuNRs in the absence of bacteria, and $N_{\text{bacteria}}$ corresponds to the count of AuNRs with the involvement of bacteria.

### 2.12 Ethics statement

Ethical approval for this study was obtained from the Ethics Committee at Zhongshan Hospital Affiliated to Fudan University (B2022-044R, approved February 25, 2022). *E. coli* expressing Klebsiella pneumoniae carbapenemase 2 (*E. coli* expressing KPC-2) were obtained from previously collected and anonymized routine microbiological specimens, no patients were directly involved in this study.

## 3 Result and discussion

### 3.1 Preparation and characterization of alkyne–modified AuNRs and azide–functionalized coverslip

**Fig. 1:**
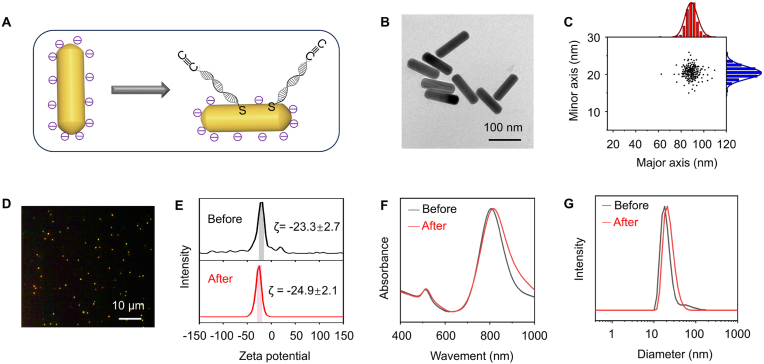
Alkyne-functionalized gold nanorods (AuNRs) were characterized as follows: (A) A schematic illustrates citrate-modified AuNRs before and after alkyne-DNA conjugation. (B) and (C) Transmission electron microscopy (TEM) images and size distributions (long and short axes) of citrate-modified AuNRs. (D) DFM images depict AuNRs following the addition of sodium ascorbate (NaAsc, 100 μM), Cu^II^ (10 μM), and alkyne-functionalized AuNRs to azide-functionalized coverslips. (E) Zeta potential distributions are presented for citrate-modified AuNRs before and after alkyne-DNA modification. (F) UV–vis absorbance spectra of citrate-modified AuNRs before and after alkyne-DNA modification. (G) Hydration radius distributions of citrate-capped AuNRs before and after alkyne-DNA modification. (For interpretation of the references to color in this figure legend, the reader is referred to the Web version of this article.)

Anisotropic gold nanorods (AuNRs), acting as one-dimensional nanomaterials, exhibit polarization-dependent colorful dark-field scattering [42]. In typical experiments, individual AuNRs (CTAB-modified or citrate-modified AuNRs) were selected as the imaging probes for DFM. The alkyne and thiol-modified DNA oligonucleotide strands first hybridized with the complementary strands to form a rigid double-stranded structure (dsDNA), which was subsequently assembled on the surface of AuNRs by the formation of Au–S bonds (Fig. 1A, Fig. S1A; DNA sequences are shown in Table S2). For alkyne–modified CTAB-modified AuNRs, the transmission electron microscopy (TEM) images are shown in Fig. S1B. The prepared alkyne–modified AuNPs had a homogeneous rod-like structure with a long diameter of 80 nm and a short diameter of 20 nm (Fig. S1C). Likewise, alkyne-functionalized citrate-modified AuNRs have a long diameter of 90 nm and a short diameter of 22 nm (Fig. 1B and C). The modification process of alkyne–modified AuNRs by dsDNA could first be monitored by changing the zeta potentials of AuNRs (Figs. S3 and 1E). The zeta potential of the CTAB-modified AuNRs shifted from +39.1±3.1 to +22.5±2.9 following the modification of alkyne–dsDNA (Fig. S3), suggesting that the CTAB ligand was successfully replaced by alkyne–dsDNA and partially neutralized some of the positive charges. For the sodium citrate-modified AuNRs, the zeta potential exhibited negligible changes before and after the modification with alkyne–dsDNA (Fig. 1E), which could be attributed to the negative charges carried by both citrate and double-stranded DNA. Moreover, the modification of alkyne–modified AuNRs by dsDNA could be easily monitored by the red-shifted surface plasmon resonance absorption peaks (Fig. 1F and S4) and the increased hydrodynamic sizes (Fig. 1G and S5).

**Fig. 2:**
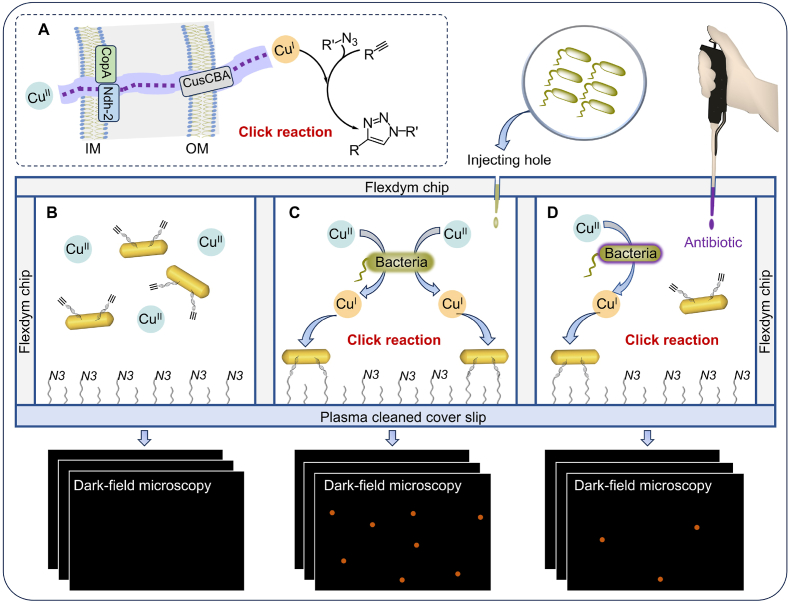
Conceptual illustration of the bacteria-initiated click chemistry for POC microbial detection. (A) Depicts relevant copper homeostasis mechanisms in *E. coli* and the copper(I)-catalyzed azide-alkyne cycloaddition (CuAAC) reaction. Specifically, CopA (Cu^I^-translocating P-type ATPase), Ndh-2 (cupric reductase), and CusCBA (copper efflux pump) [45]. (B), (C), and (D) present schematic representations and DFM images within the microreactor for three scenarios: (B) absence of bacteria, (C) presence of bacteria, and (D) presence of both bacteria and antibiotics.

Passivation and modification of the coverslip surface utilizing PLL–PEG–azide was conducted with a slight modification based on previous methods [41]. In brief, the sample cells underwent an initial etching process using vacuum plasma, followed by co-incubation with PLL–PEG and PLL–PEG–azide. To confirm the successful alkyne functionalization of the coverslips, we utilized click chemistry to assess the Cu^I^-catalyzed conjugation between azide-functionalized coverslip and AuNRs functionalized with the terminal alkyne. In the presence of ascorbate sodium, the catalyst (Cu^I^) was conveniently derived from the reduction of Cu^II^. The generation of scattered signals from AuNRs was observed via DFM (Fig. 1D and S2). However, the CuAAC reaction could not occur when only the PLL–PEG modification was applied to the coverslip slides. Subsequently, no significant scattering signal of individual plasmonic nanoparticles was observed via DFM (Fig. S6).

### 3.2 Principle of the AuNRs–BICC method for POC microbial detection

Copper, a redox-active transition metal, is crucial for aerobic metabolism, necessitating tight control of intracellular copper homeostasis. Under anaerobic conditions, copper undergoes a transition from the Cu^II^ to the Cu^I^ oxidation state. Consequently, intracellular copper concentrations are maintained within very narrow physiological limits [[43], [44], [45]]. This is primarily due to the ability of Cu^I^ to readily diffuse across the cytoplasmic membrane, facilitating its role as a selective catalyst for alkyne-azide cycloaddition reactions. Initially, we conceived and fabricated a micro-upgrade (30 μL) sterile and closed microreactor conjugated by one azide-functionalized coverslip with two punched Flexdym chips. In the presence of bacteria, Cu^II^ can be reduced to Cu^I^ by autologous copper-binding and the pathway (Fig. 2A), which results in the attachment of alkyne-functionalized AuNRs to the sensing interfaces (azide–functionalized coverslip) via a Cu^I^-catalyzed azide–alkyne cycloaddition (CuAAC) reaction [[46], [47][45], [46]]. In the presence of bacteria, the number of bacteria was estimated by counting the quantity of AuNRs attached to the sensing interface via the CuAAC reaction via DFM (Fig. 2C). The CuAAC reaction could not occur in the absence of bacteria and was used as the control. No observable counts of AuNRs were detected via DFM (Fig. 2B). Following the interaction of antibiotics with bacteria, the bacterial activity was inhibited, resulting in a diminished reduction ability of Cu^II^ to Cu^I^. This reduction in reactivity diminished the efficiency of the CuAAC reaction, ultimately leading to a decrease in the count of AuNRs observed via DFM (Fig. 2D). Consequently, this sensor is expected to function as an indicator of bacterial viability and serve as a valuable tool for drug screening.

### 3.3 Bacteria-instructed click chemistry between the azide–functionalized coverslip and alkyne–modified AuNRs

**Fig. 3:**
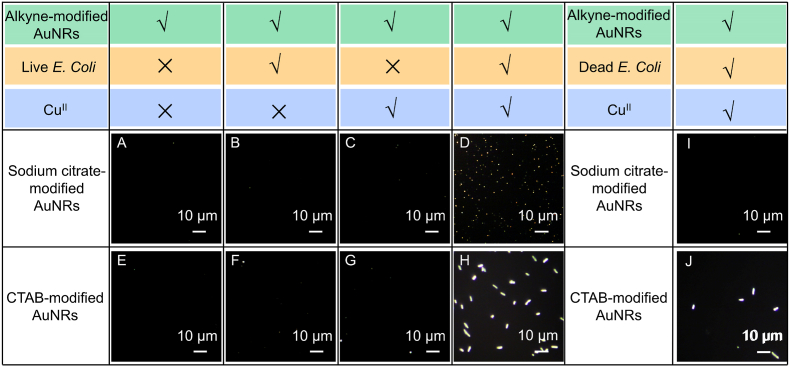
DFM images were acquired under various conditions. For citrate-modified AuNRs: (A) AuNRs only; (B) AuNRs without Cu^II^; (C) AuNRs without live *E. coli*; (D) AuNRs with Cu^II^ and live *E. coli*; (I) AuNRs with Cu^II^ and dead *E. coli*. For CTAB-modified AuNRs: (E) AuNRs only; (F) AuNRs without Cu^II^; (G) AuNRs without live *E. coli*; (H) AuNRs with Cu^II^ and live *E. coli*; (J) AuNRs with Cu^II^ and dead *E. coli*.

To investigate the feasibility of bacteria-instructed click chemistry of individual AuNRs on the azide–functionalized coverslip, a model bacterium, *E. coli*, with a concentration of 10^7^ colony-forming units, was introduced into the microreactor and mixed with alkyne–modified AuNRs in the presence of Cu^II^ on the azide–functionalized coverslip. As expected, as shown in Fig. 3D and H, the occurrence of bright scattered signals in DFM necessitates the simultaneous presence of the azide–functionalized coverslip, alkyne–modified AuNRs, and Cu^II^ following 2 h of incubation with live *E. coli*. Indeed, as a control, the other groups (without live *E. coli*, without Cu^II^, or without live *E. coli* and Cu^II^) did not show any significant dark-field scattered signals in DFM (Fig. 3A–C, 3E–3G), which was attributed to the gold nanoparticles not participating in the Cu^I^-catalyzed click chemistry reaction and not forming covalent bonds with the substrate. In addition, bacteria were inactivated by heating at 80 °C for 10 min, ensuring complete loss of viability while maintaining structural integrity. We found the dead bacteria could not catalyze the conversion of Cu^II^ to Cu^I^ in situ. Thus, the scattered signals from plasmonic nanoparticles attached to the coverslip slide could not be monitored under DFM (Fig. 3I and J). Interestingly, there was a noticeable distinction in the scattered signals observed via DFM for the bacterial-instructed click chemistry reaction involving two kinds of AuNRs. For the citrate–modified AuNRs, the typical scattered signal of AuNRs was observed via DFM (Fig. 3D). In the case of CTAB–modified AuNRs, the scattered signal exhibited an elongated shape resembling that of *E. coli*, and these signals did not originate from the plasmonic nanorods upon DFM (Fig. 3H). In contrast, when only CTAB–modified AuNRs or *E. coli* was present, there was no noticeable scattered signal (Fig. S7). However, upon mixing *E. coli* with CTAB–modified AuNRs, a significantly enhanced intensity and area magnification of the scattered signal counts were observed (Fig. 3H, Fig. S8). This enhancement may result from electrostatic interactions, with positively charged CTAB–modified AuNRs adhering to the negatively charged bacterial surface, significantly enhancing the scattered signal from the bacteria (as illustrated in Fig. S9). Therefore, we utilized negatively charged citrate–modified AuNRs in subsequent experiments as single-particle dark-field imaging probes.

### 3.4 Sensing performance of the AuNRs–BICC method for the quantification of bacteria

**Fig. 4:**
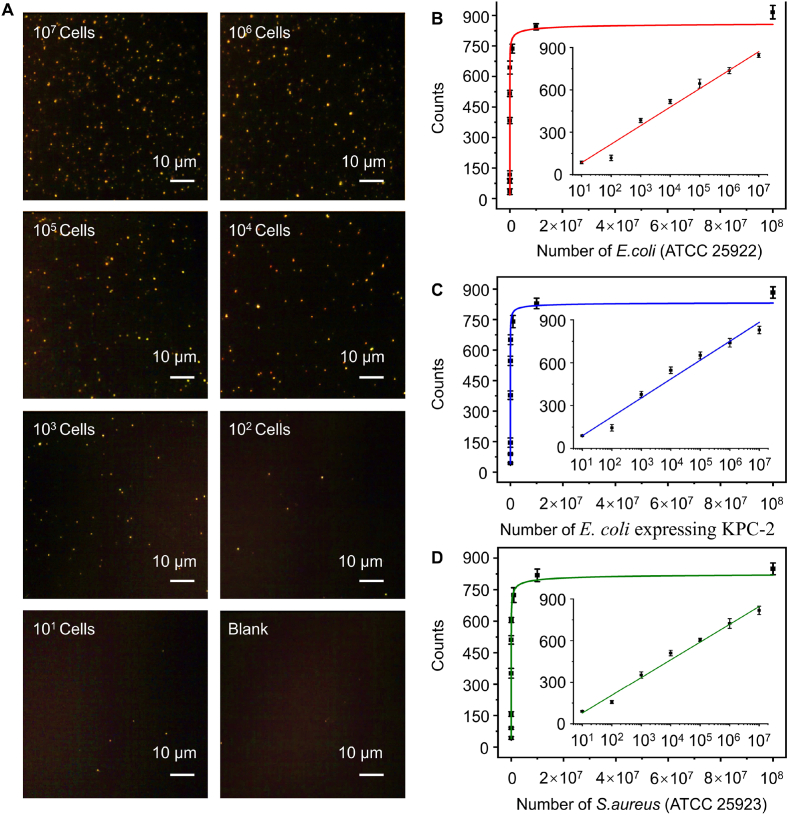
Bacteria assay sensitivity: (A) DFM images of the AuNRs corresponding to 0, 10^1^, 10^2^, 10^3^, 10^4^, 10^5^, 10^6^, and 10^7^ cells of *E. coil*. (B) Graph of the counts of AuNR changes with different quantities of *E. coli* from 10^1^ to 10^8^ cells. The inset shows the linear relationship between the AuNR counts and the logarithm value of the number of *E. coli*. The graphs in (C) and (D) display the variation in the counts of AuNRs as they change with different quantities of *S. aureus* and *E. coli* expressing KPC-2, respectively, showing a linear relationship in each inset.

To examine the sensing performance of the single-particle counting strategy based on Cu^I^-catalyzed click chemistry for bacteria quantification, we added different quantities of bacteria into the microreactor (azide–functionalized coverslip, 1 mg/mL alkyne–AuNRs, 10 μM of Cu^II^, 2 h of bacterial reaction time) to count the AuNRs attached to the sensing interface. The average counts of AuNRs in the eight regions were extracted for enumeration to minimize measurement errors. Fig. 4A shows the DFM images of AuNRs in the presence of different quantities of *E. coil* (0 to 10^7^ cells). As the quantities of *E. coli* increased, the counts of AuNRs also increased rapidly, tending to reach saturation upon further increasing the quantities of *E. coli* beyond 10^7^ cells (Fig. 4B). In the range of *E. coli* from 10^1^ to 10^7^ cells, the counts of AuNRs and the logarithm of the quantities of *E. coli* showed a good linear relationship. As shown in the inset of Fig. 4B, the linear regression equation is $C=131\;\log_{10}\;N-46.7$, ($R^{2}=0.982$), where C is the net counts of AuNRs, and N is the number of bacteria. Furthermore, we confirmed the versatility of the single-particle counting strategy with two other types of bacteria: gram-positive *S. aureus* and antibiotic-resistant *E. coli* strains (*E. coli* expressing KPC-2). As depicted in Fig. 3C and D, we also obtained the linear calibration curves for *S. aureus* ($C=132\;\log_{10}\;N-43.5$, $R^{2}=0.988$) and *E. coli* expressing KPC-2 ($C=128\;\log_{10}\;N-45.0$, $R^{2}=0.987$) relating the net counts of AuNRs to the number of bacteria from 10^1^ to 10^7^ cells. The differences in slope observed in the linear calibration curves obtained for various pathogenic bacteria may be attributed to their distinct capacities for reducing Cu^II^ [14,24,[45], [46]]. The limit of detection (LOD) values were defined as the minimum observable bacterial concentration that could be reliably detected under the experimental conditions. And this AuNRs–BICC method exhibited sensitivity down to 10^1^ cells for the quantitative detection of bacteria while maintaining an extended linear range from 10^1^ to 10^7^ cells, along with a simplified optical imaging setup. This may be explained by the controllable fixation of PLL–PEG–azide on the surface of the coverslip, the high efficiency of bacteria in reducing Cu^II^ to Cu^I^ [47,48], the specificity and high efficiency of the Cu^I^-catalyzed azide–functionalized coverslip and the alkyne–modified AuNRs cycloaddition promoted by ring strain, and the low background of DFM.

### 3.5 Portable platform for detecting bacteria in complicated sepsis blood samples

**Fig. 5:**
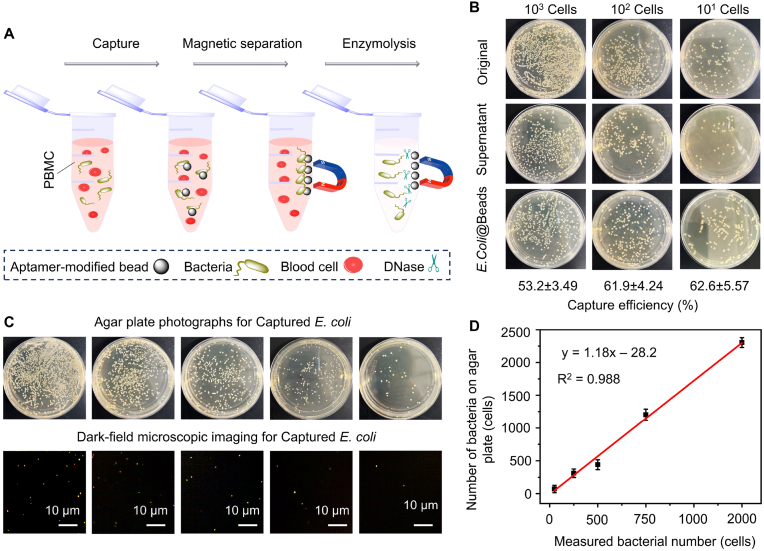
(A) Schematic illustration of magnetic capture and separation by Fe_3_O_4_ magnetic nanoparticles modified with bacterial species-identifiable aptamers from the blood sample and enzymolysis by DNase 1. (B) Capture efficiency of SA–MBs coupled with the 3′-biotin–*E. coli* aptamer toward *E. coli* bacteria with concentrations ranging from 10^1^, 10^2^, and 10^3^ cells after 30 min of incubation. (C) Agar plate cultivation images and AuNRs–BICC images of the same quantity of bacteria obtained upon magnetic separation and DNase-triggered release. (D) Linear correlation analysis between the determined number of bacteria in the blood sample using the AuNRs–BICC method and agar plate cultivation.

Beyond general bacterial detection, the ability to selectively identify and quantify specific bacterial strains within complex real-world samples holds significant clinical importance [49]. To detect one specific bacterial strain coexisting with other strains, we first constructed one aptamer-assisted bacterial capturer and then quantified the number of bacteria using the AuNRs–BICC method. This bacterial separation system is based on Fe_3_O_4_ magnetic nanoparticles modified with bacterial species-identifiable aptamers to realize separation and enrichment of specific bacterial strains from a blood sample (the sequences of oligonucleotides for *E. coil* and *S. aureus* provided in Table S2, Fig. S10, and Fig. S11). As demonstrated in Fig. 5A, the concentrated bacteria were released from magnetic nanoparticles via treatment with DNase 1 following magnetic separation. The released bacteria reduced Cu^II^ to Cu^I^, subsequently catalyzing the CuAAC reaction. Quantitative determination of bacteria can be achieved by counting the AuNRs using DFM. As illustrated in Fig. 5B, when *E. coli* was captured using SA–MBs coupled with the 3′-biotin–*E. coli* aptamer, the capture efficiency exceeded 50 % (*E. coli* cells ranging from 10^1^ to 10^3^) within 30 min. However, when carboxyl-functionalized magnetic beads were covalently modified with amino-functionalized aptamers, the efficiency of capturing *E. coli* was only 20 % (Fig. S12). We speculate that the higher capture efficiency is partly attributed to the stronger affinity between biotin and streptavidin (Kd ≈ 10^−14^ M) [50]. Additionally, the binding of biotin and streptavidin on the surface of the magnetic beads provides the oligonucleotides with greater stretching space, which is more conducive to capturing bacteria. Similarly, when the surface of SA–MBs was modified with *S. aureus*-specific aptamers, the efficiency of capturing *S. aureus* approached 60 % (Fig. S13).

After magnetic separation and DNase-triggered release, the concentrated *E. coli* obtained from the complex sepsis blood samples was mixed with alkyne–modified AuNRs and Cu^II^ and then introduced into the microreactor. To validate the feasibility of our method, we compared the results obtained from the AuNRs–BICC method with the culture method using *E. coli* as the model. Equivalent bacteria obtained via magnetic separation and DNase-triggered release were divided into two parts. One was plated onto agar plates for cultivation, while another was introduced into the click chemistry reactor (Fig. 5C). As shown in Fig. 5D, the number of bacteria measured using the AuNRs–BICC method closely matches the number obtained from the culture method. Compared to prolonged bacterial agar plate cultivation (>24 h), the entire process of bacterial magnetic enrichment and separation, release triggered by DNase 1, and single-particle enumeration can be accomplished in 3 h, meeting the time constraints of clinical POC sensing.

### 3.6 Evaluation of bacterial AST using single-particle click chemistry

**Fig. 6:**
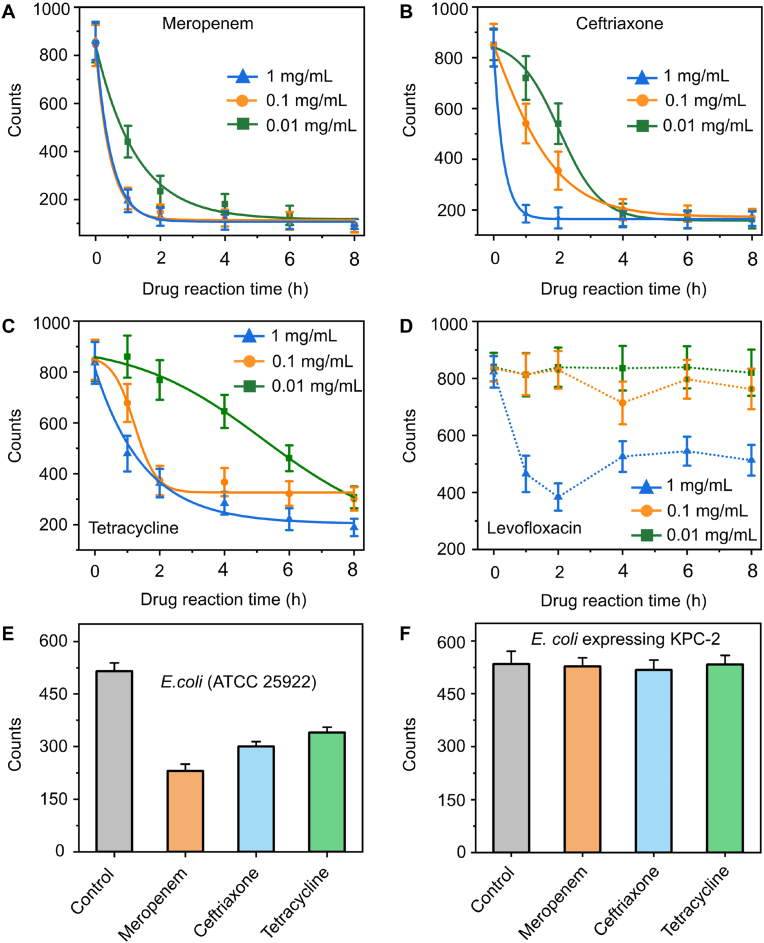
AST responses of *E. coli* (10^8^ CFU/mL) to different antibiotics for different periods: (A) meropenem, (B) ceftriaxone, (C) tetracycline, and (D) levofloxacin, assayed by the AuNRs–BICC method. The antibiotic concentrations were 0.01, 0.1, and 1 mg/mL, with error bars representing the standard deviation (n = 3). AST responses of (E) *E. coli* (ATCC 25922) (5000 cells) and (F) *E. coli* expressing KPC-2 (5000 cells) when bacteria were co-cultured with four antibiotics for 90 min.

Antibiotic susceptibility testing (AST) of *E. coli* was performed using the AuNRs-BICC method to assess the impact of various antibiotics on bacterial viability. Antibiotic susceptibility testing (AST) was performed using four antibiotics with distinct mechanisms of action: meropenem, ceftriaxone, tetracycline, and levofloxacin. Meropenem and ceftriaxone are β-lactam antibiotics that inhibit bacterial cell wall synthesis. Tetracycline, a tetracycline antibiotic, inhibits protein synthesis. Levofloxacin, a fluoroquinolone antibiotic, inhibits DNA replication and transcription. Bacterial suspensions (10^8^ cells) were incubated with the antibiotics at different concentrations (0.01, 0.1, and 1 mg/mL) for different periods and then mixed with alkyne–modified AuNRs and Cu^II^ onto the azide–functionalized coverslip in a microreactor for AuNRs–BICC detection. As depicted in Fig. 6, the AST responses of *E. coli* to different antibiotics varied. In the presence of *β*-lactam antibiotics, *E. coli* viability decreased sharply, with meropenem exhibiting the strongest inhibitory effect across all three concentrations (Fig. 6A and B). At a concentration of 0.01 mg/mL, tetracycline displayed mild inhibitory effects (Fig. 6C), while levofloxacin, even at three different concentrations, failed to completely deactivate all bacterial cells after 8 h of incubation (Fig. 6D). Additionally, we confirmed the reliability of these results using the bacterial culture method. As shown in Fig. S14, the bactericidal effects of meropenem, ceftriaxone, and tetracycline measured by the bacterial culture method were consistent with those observed using the AuNRs–BICC method.

Unexpectedly, the AST results for *E. coli* exposed to levofloxacin, as determined by the AuNRs–BICC method, differed from those obtained using the bacterial culture method. The results from the bacterial culture method indicated that levofloxacin at high concentrations effectively inhibited bacterial growth. In contrast, the AuNRs–BICC method showed that even at the highest concentration of levofloxacin, the rates of click chemistry reactions did not decrease significantly. Similar findings were reported by Mason et al. [51], in which observed that the bacteria remained viable but entered a non-proliferative state after incubation with high concentrations of quinolone antibiotics, continuously reducing Cu^II^ to Cu^I^. Therefore, we did not observe a reduction in the counts of AuNRs, even at high concentrations of levofloxacin. This is a limitation of the AuNRs–BICC method is that it is not suitable for screening quinolone antibiotics.

We also conducted AST on two bacterial strains, drug-sensitive *E. coli* and *E. coli* expressing KPC-2, exposed to three antibiotics (meropenem, ceftriaxone, and tetracycline). As illustrated in Fig. 6E, the activity of *E. coli* was inhibited by the antibiotics, resulting in a decrease in the counts of AuNRs detected by the AuNRs–BICC method. In contrast, *E. coli* expressing KPC-2, as one carbapenem-resistant Enterobacteriaceae (CRE), displayed resistance to all three antibiotics, and consequently, the counts of AuNRs detected by the AuNRs–BICC method remained relatively unchanged (Fig. 6F). As expected, the results obtained from the AuNRs–BICC method were consistent with the results obtained from the bacterial culture method (Fig. S15).

## 4 Conclusion

In summary, this work presents a novel microbial sensor (AuNRs–BICC) based on citrate- and alkyne-modified gold nanorods (AuNRs) with click chemistry properties and exceptional biocompatibility. These AuNRs enable a unique approach for bacterial quantification, viability assessment, and antimicrobial susceptibility testing. The sensor harnesses the bacterial metabolic process for Cu^II^ binding and reduction, triggering a click chemistry reaction between azide-functionalized coverslip surfaces and alkyne-modified AuNRs. This reaction is visualized through dark-field microscopy imaging and analyzed via single-particle enumeration with bacteria-initiated click chemistry. The AuNRs–BICC sensor offers several advantages: (i) high efficiency and excellent biocompatibility, (ii) a clean imaging background without nonspecific scattering signals, (iii) simplicity and portability in detection and preparation, and (iv) rapid (within 3 h) and sensitive bacterial quantification (down to 10^1^ cells), viability assessment, and antimicrobial susceptibility testing. Compared to traditional methods, this AuNRs–BICC sensor shows great potential for clinical point-of-care (POC) diagnosis of bacterial infections, evaluating the effectiveness of antibacterial drugs, and helping to reduce the occurrence of bacterial resistance.

## CRediT authorship contribution statement

**Tongtong Tian:** Writing – original draft, Visualization, Project administration, Methodology, Funding acquisition, Conceptualization. **Wenjing Yang:** Methodology, Investigation, Formal analysis, Data curation, Conceptualization. **Xiaohuan Wang:** Methodology, Investigation, Formal analysis, Data curation. **Te Liu:** Investigation, Formal analysis, Data curation. **Baishen Pan:** Investigation, Formal analysis, Data curation. **Wei Guo:** Writing – review & editing, Validation, Funding acquisition. **Beili Wang:** Writing – review & editing, Validation, Supervision, Resources.

## Declaration of competing interest

The authors declare that they have no known competing financial interests or personal relationships that could have appeared to influence the work reported in this paper.

## Data Availability

No data was used for the research described in the article.
