# Supplementary material for: Click chemistry-enabled gold nanorods for sensitive detection and viability evaluation of copper(II)-reducing bacteria
Source: Mater Today Bio. 2025 Jan 4;30:101453. doi: 10.1016/j.mtbio.2025.101453 (PMC11764086; doi:10.1016/j.mtbio.2025.101453)
Supplement: Multimedia component 1 [file mmc1.docx]

Supplementary Material

**Click Chemistry-Enabled Gold Nanorods for Sensitive Detection and** **Viability Evaluation of Copper(II)-Reducing Bacteria**

Tongtong Tian ^a,‡^, Wenjing Yang ^a,‡^, Xiaohuan Wang ^a,‡^, Te Liu ^e^, Baishen Pan ^a^, Wei Guo ^a,b,c,d,^*, and Beili Wang ^a,^*

^a^ *Department of Laboratory Medicine, Zhongshan Hospital, Fudan University,* *136 Yi Xue Yuan Road, Shanghai, 200032, PR China.*

^b^ *Department of Laboratory Medicine, Shanghai Geriatric Medical Center, Shanghai, PR China.*

^c^ *Department of Laboratory Medicine, Wusong Central Hospital, Baoshan District, Shanghai, PR China.*

^d^ *Department of Laboratory Medicine, Xiamen Branch, Zhongshan Hospital, Fudan University, PR China.*

^e^ *Shanghai Geriatric Institute of Chinese Medicine, Shanghai University of Traditional Chinese Medicine, No.725 South Wan Ping Road, Shanghai, 200031, PR China*

^‡^ These authors contributed equally to this work.

* Corresponding authors:

E-mail addresses: [wang.beili1@zs-hospital.sh.cn](mailto:wang.beili1@zs-hospital.sh.cn); [guo.wei@zs-hospital.sh.cn](mailto:guo.wei@zs-hospital.sh.cn)

Table of Contents

[**1.** **Material and methods** 3](#_Toc182577354)

[*1.1. Coverslips preprocessing* 3](#_Toc182577355)

[*1.2. Cultivation of several common bacteria* 3](#_Toc182577356)

[*1.3. Synthesis of the CTAB-modified AuNRs* 3](#_Toc182577357)

[*1.4. Modification of COOH-MBs with NH_2_-aptamers* 4](#_Toc182577358)

[*1.5. Modification of SA-MBs with biotinylated aptamers* 4](#_Toc182577359)

[**2.** **Figures** 5](#_Toc182577360)

[**3.** **Tables** 12](#_Toc182577361)

[**4.** **References** 14](#_Toc182577362)

1. **Material and methods**

## *1.1. Coverslips preprocessing*

The coverslips (Thermo Fisher Scientific Fisherbrand, USA, 25 mm × 25 mm) utilized in this study underwent the following cleaning procedure. First, the coverslips were subjected to ultrasonic cleaning in a 1 M potassium hydroxide (KOH) solution for 10 min, which was repeated three times. Afterward, they were rinsed in ultrapure water. Subsequently, the coverslips were ultrasonicated in ethanol for 10 min, followed by another 10 min of ultrasonication in ultrapure water, which was repeated three times for both solvents. Finally, the coverslips were dried using a stream of N_2_.

## *1.2. Cultivation of several common bacteria*

For *E. coli* (ATCC 25922), *S. aureus* (ATCC 25923), and *E. coli* expressing Klebsiella pneumoniae carbapenemase 2 (*E. coli* expressing KPC-2), the strain was placed on LB medium and continuously cultured while shaking (175 rpm) for 14 h at 37°C. The bacteria were harvested during the exponential growth phase using centrifugation. Next, the supernatant was removed, and the cell pellet was re-suspended in phosphate-buffered saline (PBS). The crude bacterial concentration could be assessed via a photometric measurement by recording the optical density (OD) at 600 nm using an APL 759 UV–vis spectrophotometer (Shanghai, China). The exact bacterial concentration was determined by enumerating colony-forming units on agar plates.

## *1.3.* *Synthesis of the CTAB-modified AuNRs*

The positive charged AuNRs were synthesized using a typical method that involves the assistance of silver ions and a seed-mediated process [1,2]. In brief, the growth solution was initially created by combining CTAB solution (17.88 mL, 0.11 M) with HAuCl_4_ (800 μL, 0.01 M). Sequentially, silver nitrate (105 μL, 0.02 M) and hydroquinone (526 μL, 0.1 M) were added while gently agitating the solution. This mixture was allowed to sit for 5 min at 30 °C within an incubator. Afterward, NaBH_4_ (34 μL, 0.498 mM) was introduced. The solution was kept at 30 °C for 12 hours in the incubator. The resulting AuNRs were subjected to two rounds of centrifugation at 10,000 rpm for 20 min each to eliminate excess reagents. After purification, the AuNRs were re-suspended in a CTAB solution (0.01 M) and stored in a brown bottle at room temperature (25 °C).

## *1.4.* *Modification of COOH-MBs with NH_2_-aptamers*

COOH-MBs and NH_2_-aptamers were combined through EDC/NHS coupling**.** In brief, 5' amino-modified *E. coli* aptamer-2 (5 nmol) was mixed with 40 µL of 0.125 M EDC/NHS in a 100 mM MES buffer at pH 4.8. The resulting mixture reached a final volume of 100 µL. 100 µL of COOH-MBs at a concentration of 10 mg/mL was then added and incubated on a roller mixer at room temperature overnight. The COOH-MBs were washed three times with 1 mL of TT buffer and resuspended in TE buffer to a concentration of 10 mg/mL.

## *1.5. Modification of SA-MBs with biotinylated aptamers*

SA-MBs and biotinylated aptamers were combined via streptavidin/biotin interaction. 5' biotin-modified aptamer (5 nmol) was mixed with 200 µL SA-MBs (10 mg/mL) and incubated on a roller mixer at room temperature for 30 min. The COOH-MBs were washed three times with 1 mL of buffer (10 mM Tris-HCl, pH 7.5, 1 mM EDTA, 1 M NaCl, 0.01% Tween-20) and resuspended in TE buffer to achieve a concentration of 10 mg/mL.

1. **Figures**

**
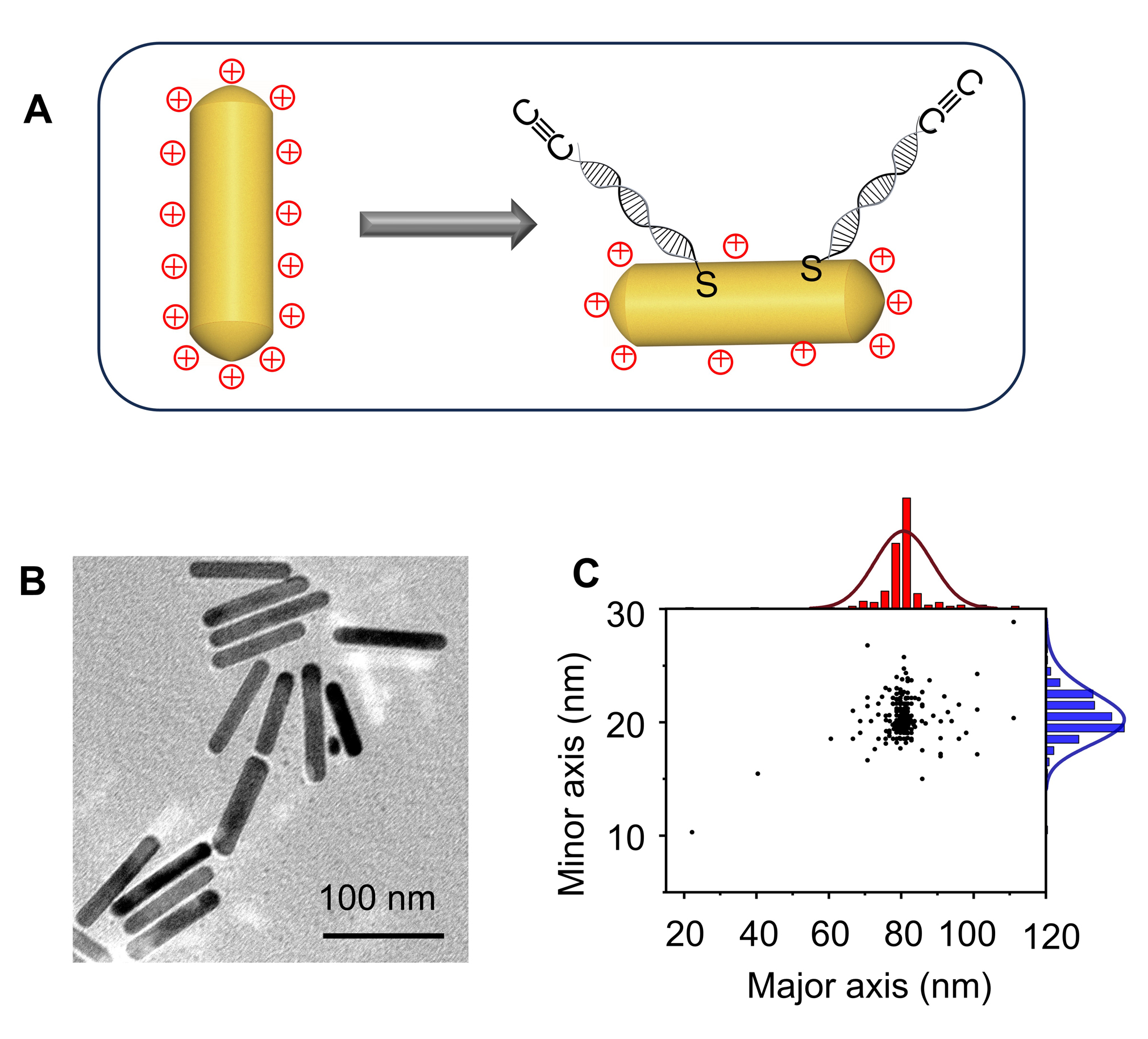
**

**Fig. S1** (A) Schematic illustration of the CTAB-modified AuNRs before and after alkyne–dsDNA modification. (B and C) TEM images and the corresponding size distribution of long and short diameters for the CTAB-modified AuNRs.

**
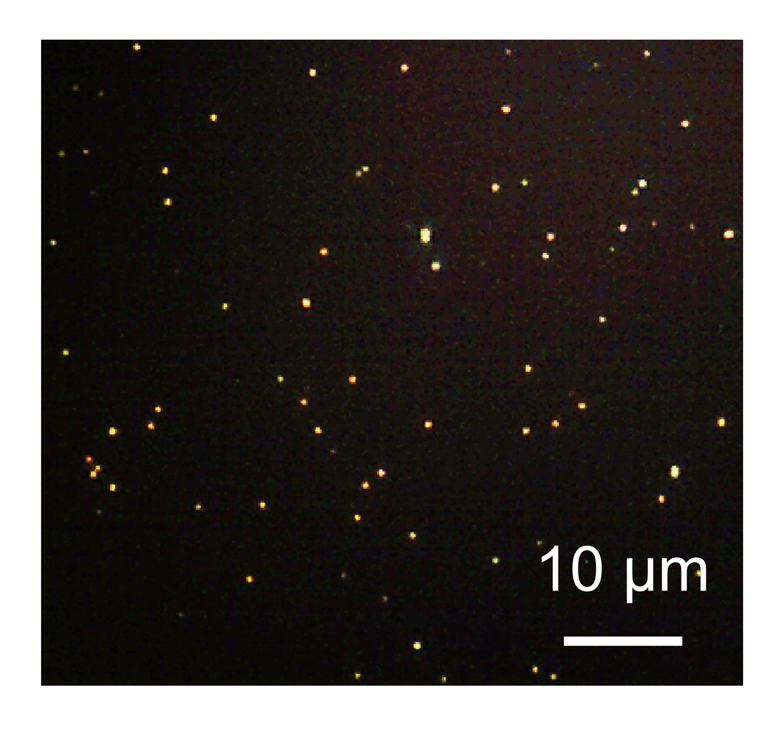
**

**Fig. S2** DFM images of AuNRs after the addition of sodium ascorbate (NaAsc, 100 μM), Cu^II^ (10 μM), and alkyne-functionalized AuNRs (CTAB-modified AuNRs) onto azide–functionalized coverslips.

**
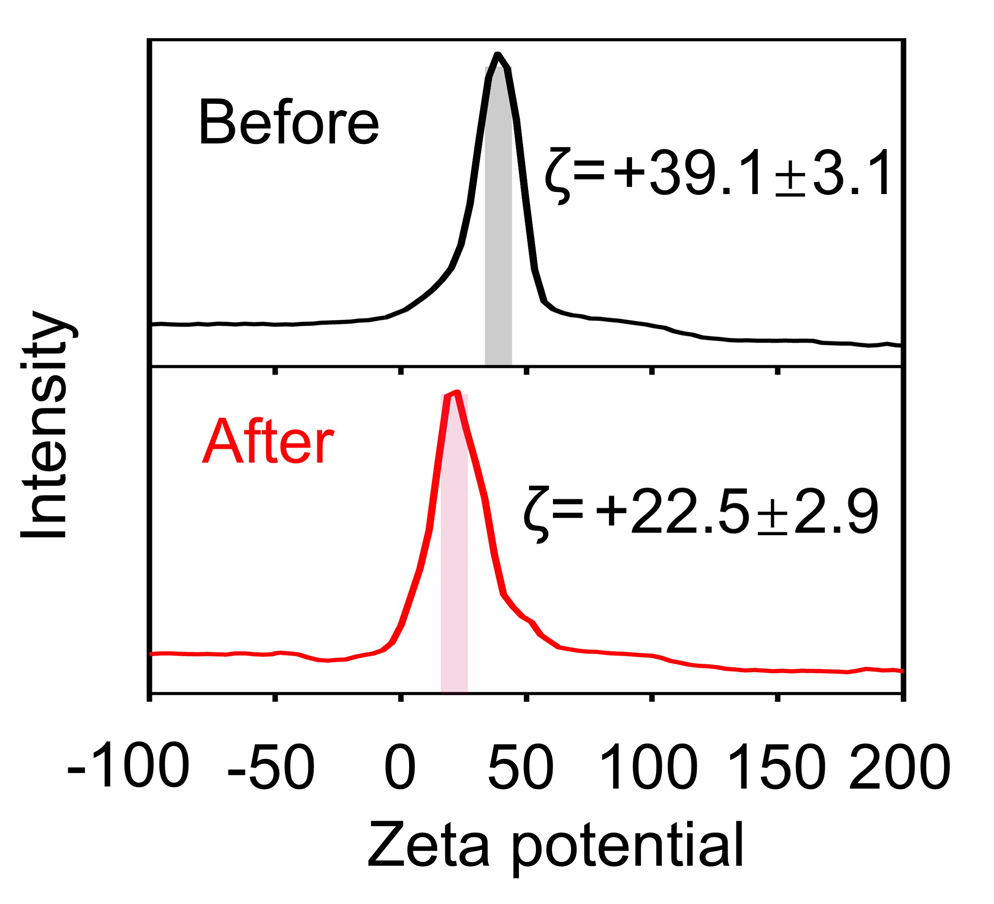
**

**Fig.S3** Zeta potential distribution of the CTAB-modified AuNRs before and after alkyne–dsDNA modification.

**
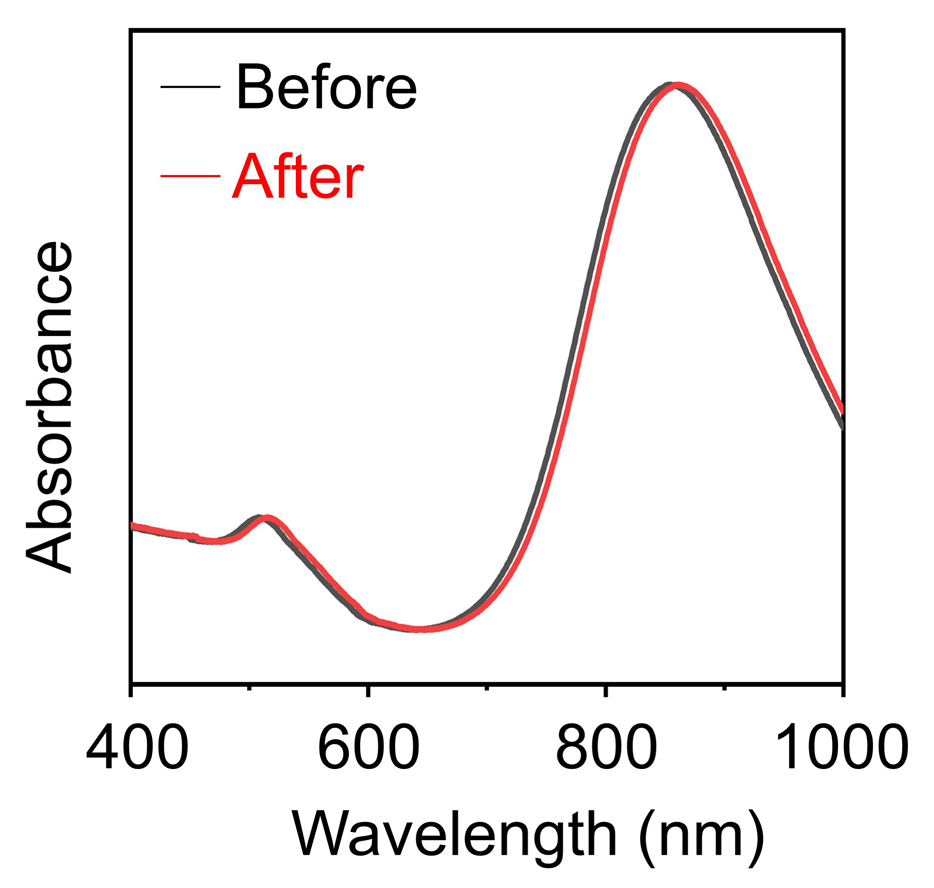
**

**Fig. S4** UV–vis absorbance spectra of the CTAB-modified AuNRs before and after alkyne–dsDNA modification.

**
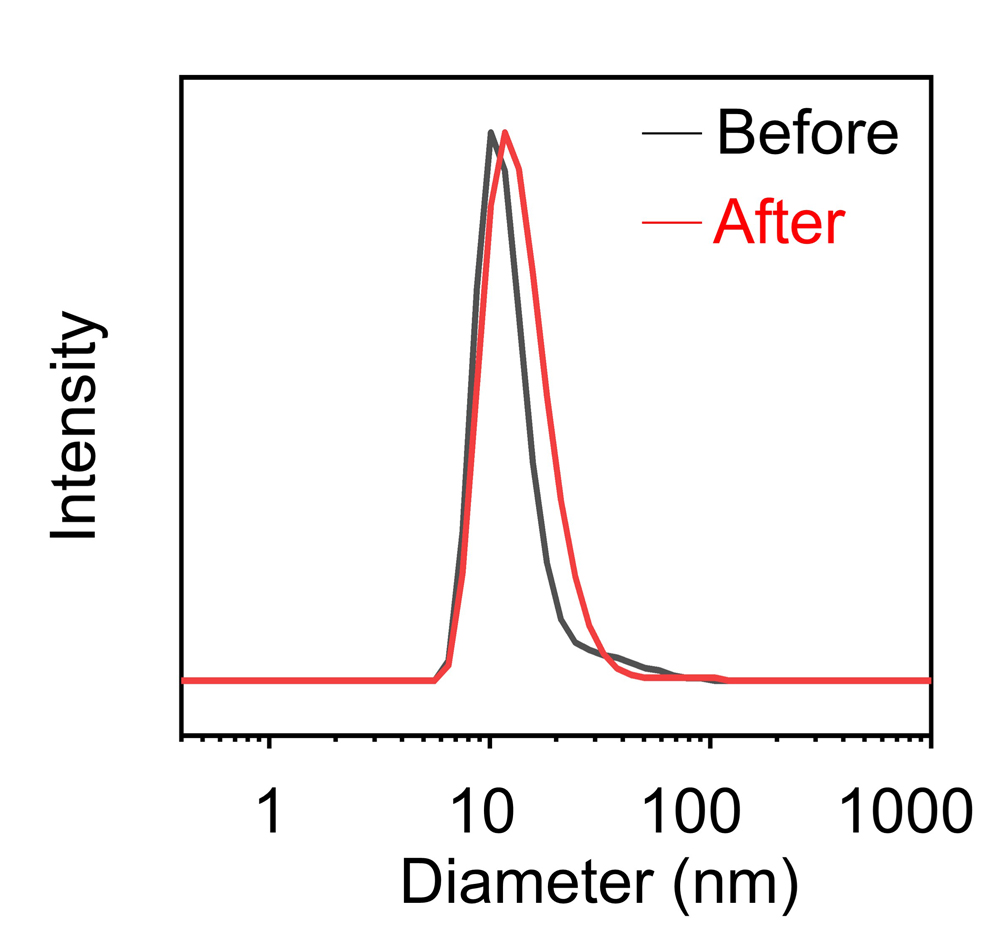
**

**Fig. S5** Hydration radius distribution of the CTAB-modified AuNRs before and after alkyne–dsDNA modification.


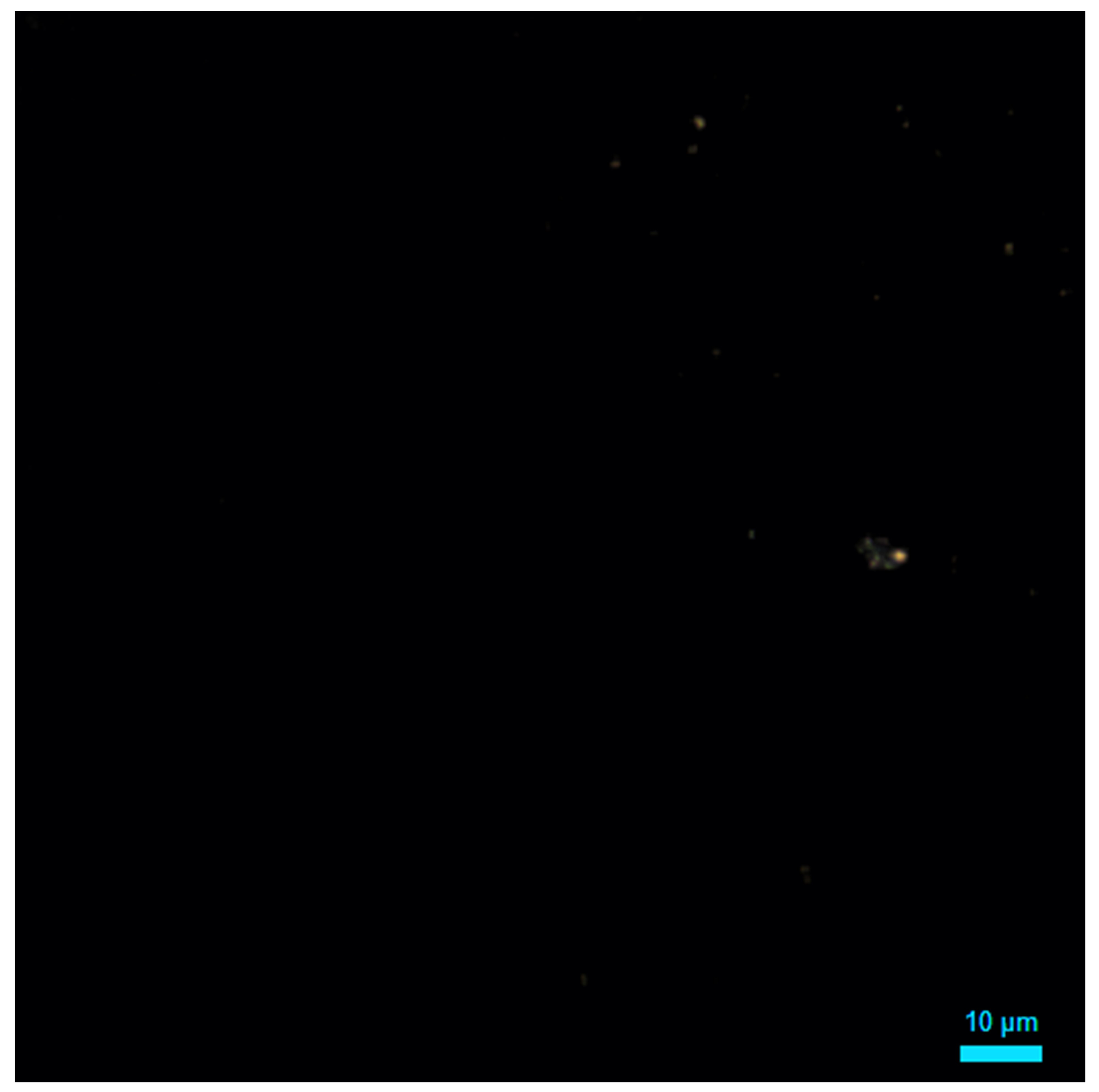


**Fig. S6** The DFM image when only PLL-PEG modification was applied to the glass slides.


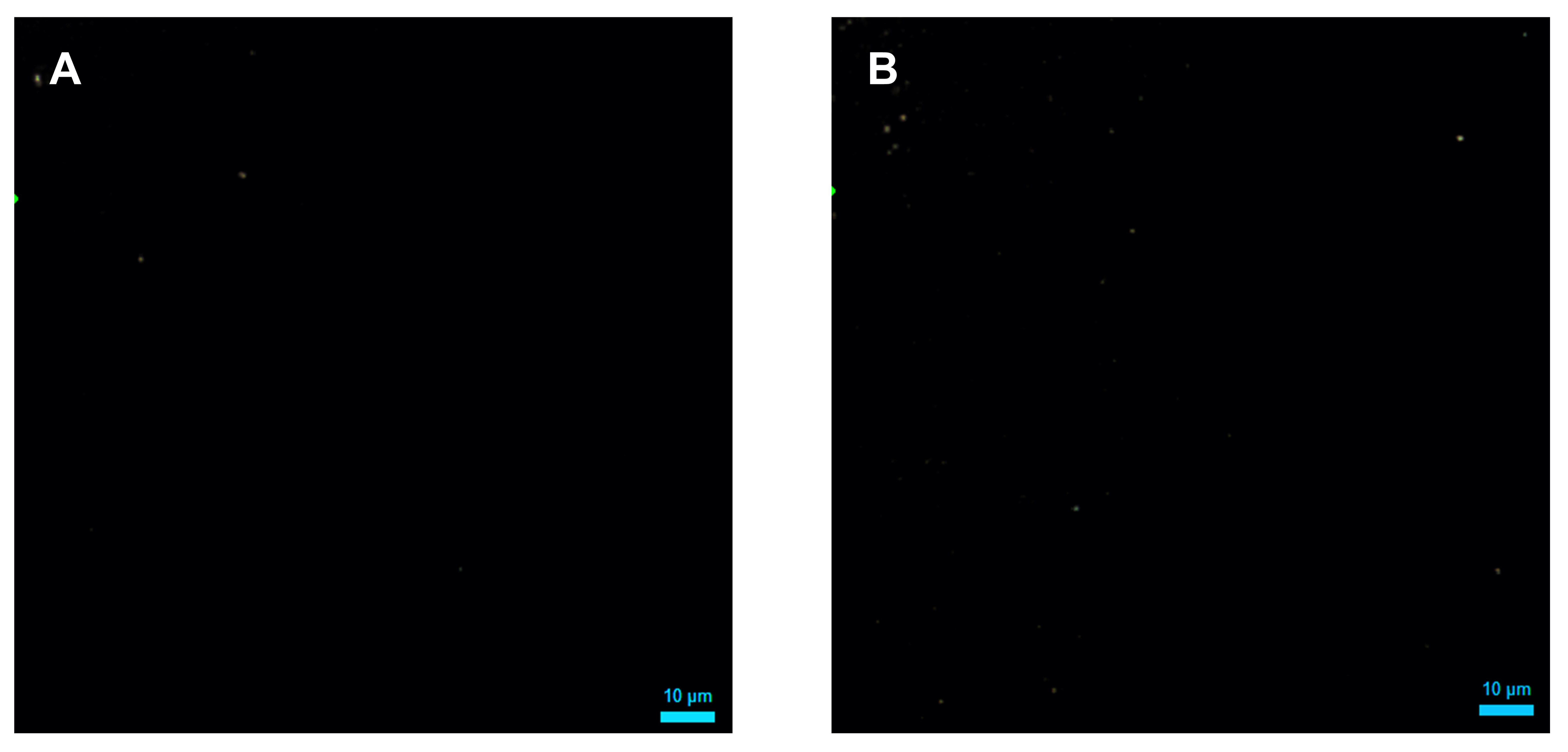


**Fig. S7** The DFM images when only CTAB-AuNRs (A) or *E. coli* (B) was present.


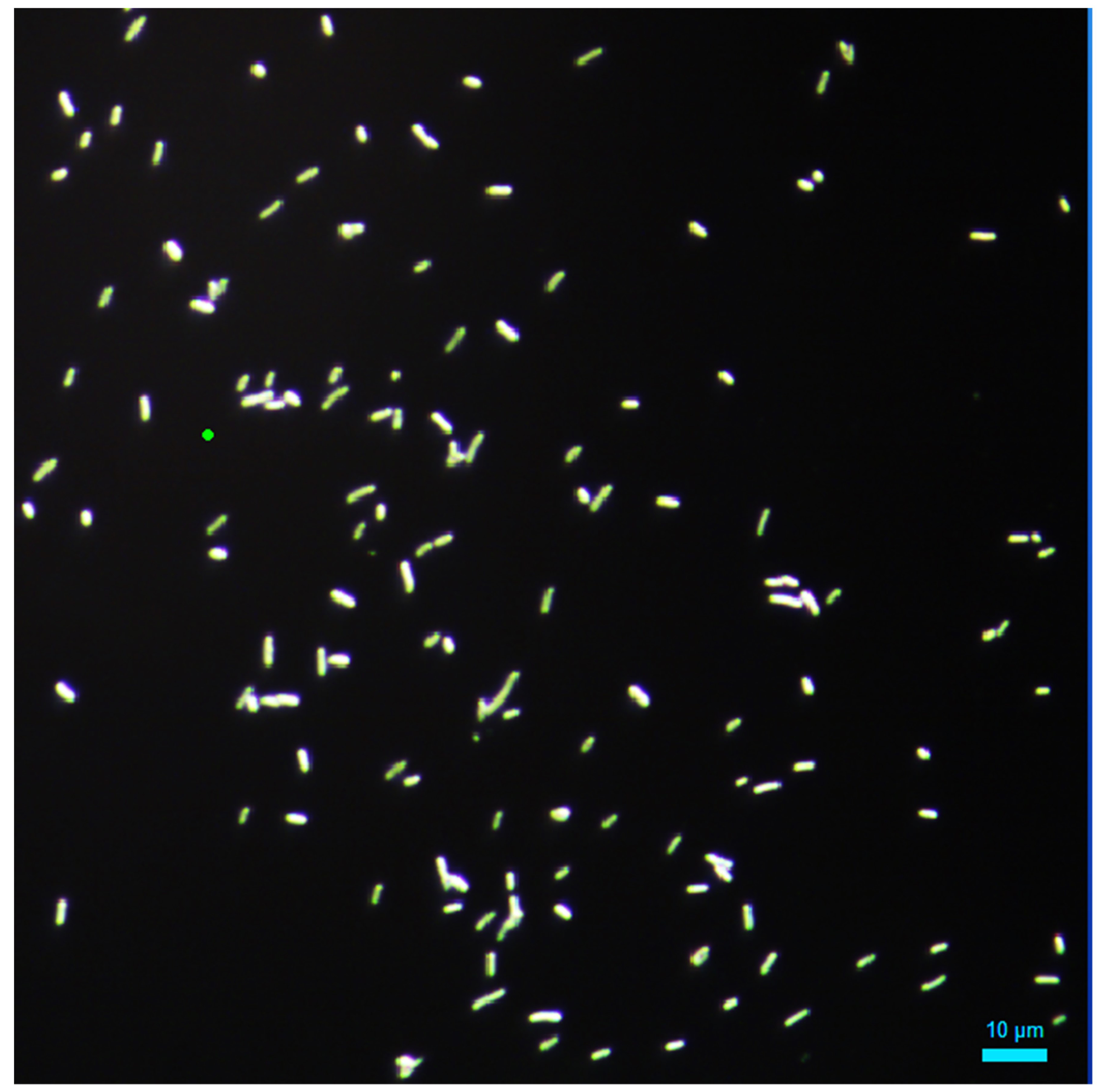


**Fig. S8** The DFM image upon mixing *E. coli* with CTAB-AuNRs.


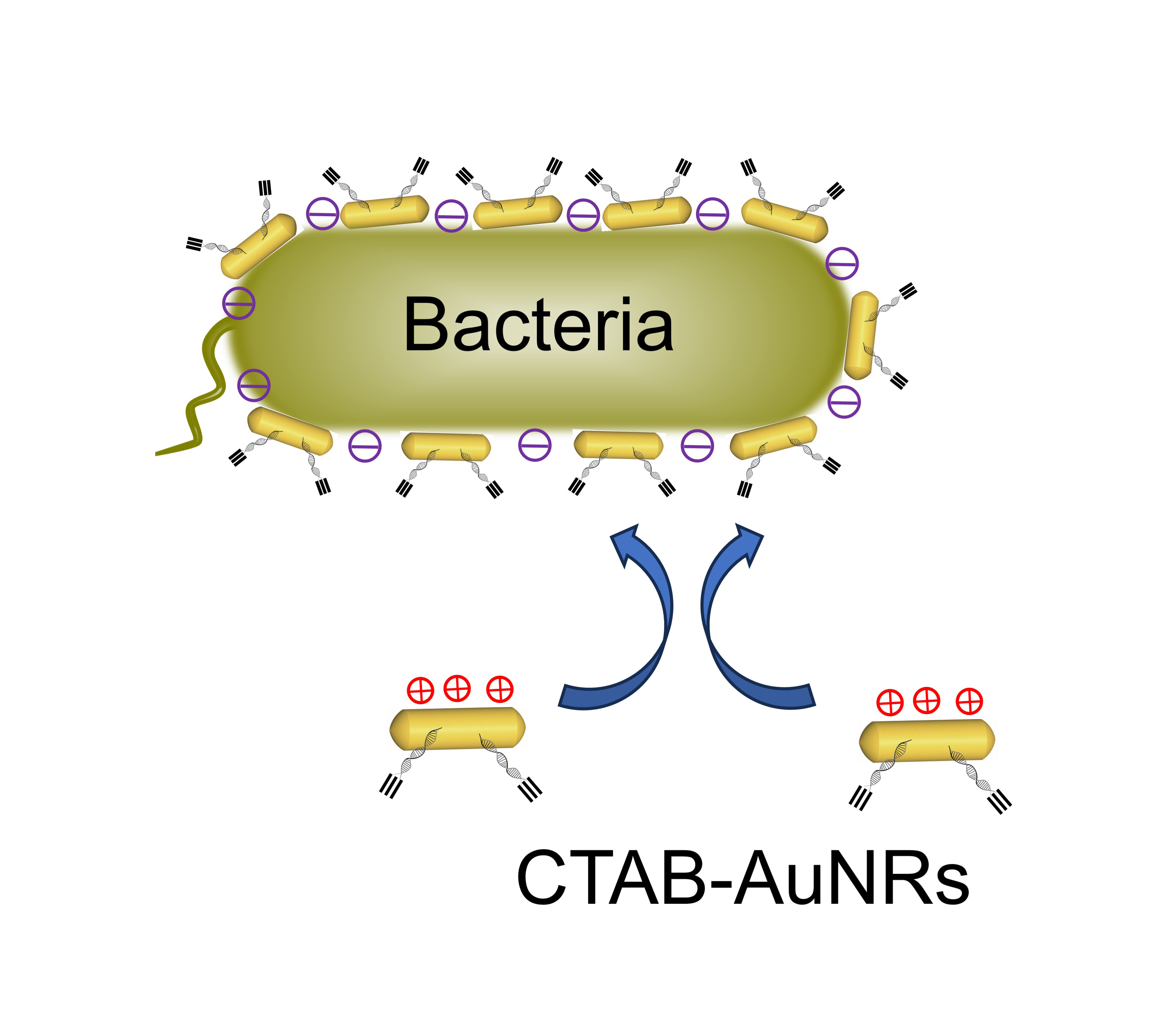


**Fig. S9** Schematic illustration of positively charged CTAB-AuNRs adhering to the negatively charged bacterial surface.


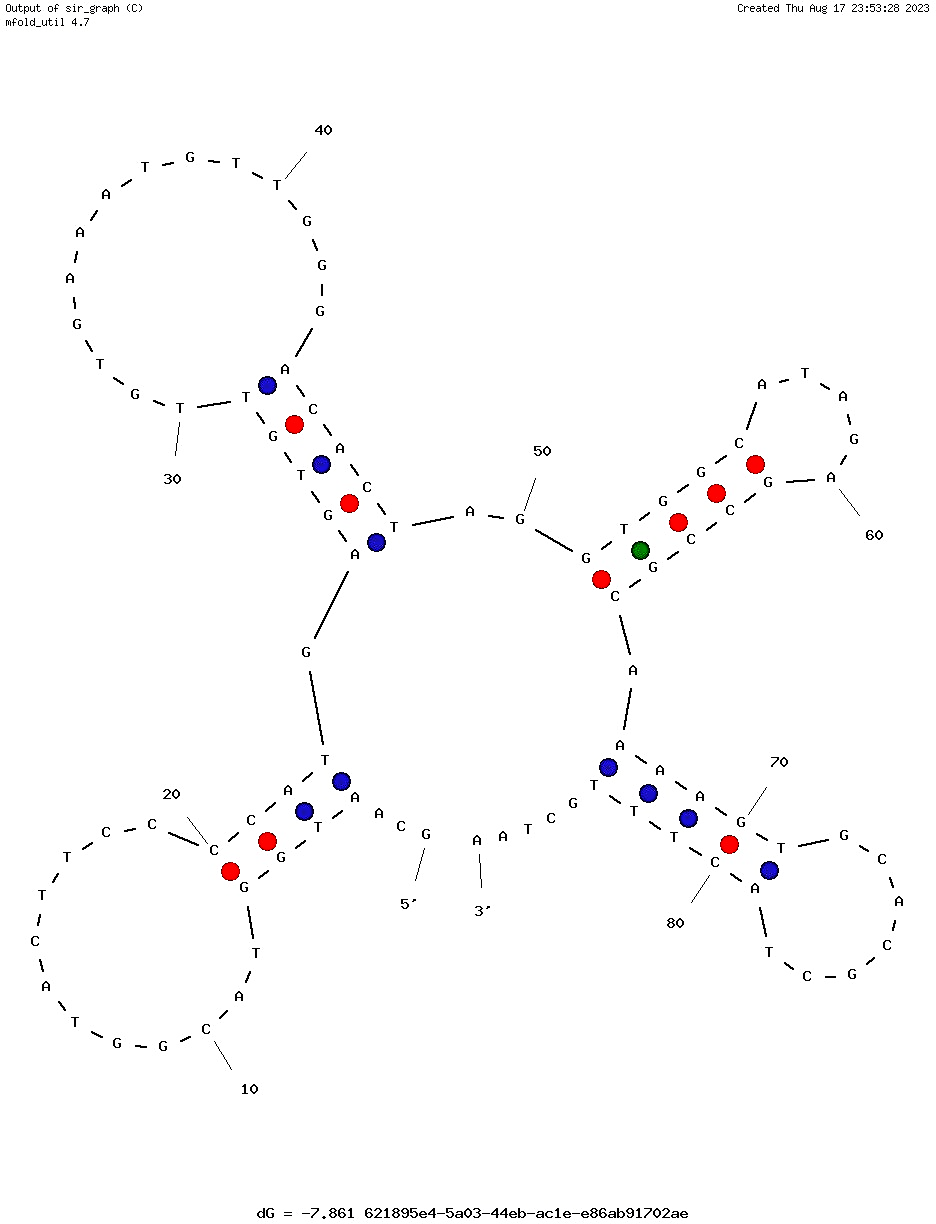


**Fig. S10** Secondary structure of *E. coli* aptamer at room temperature.


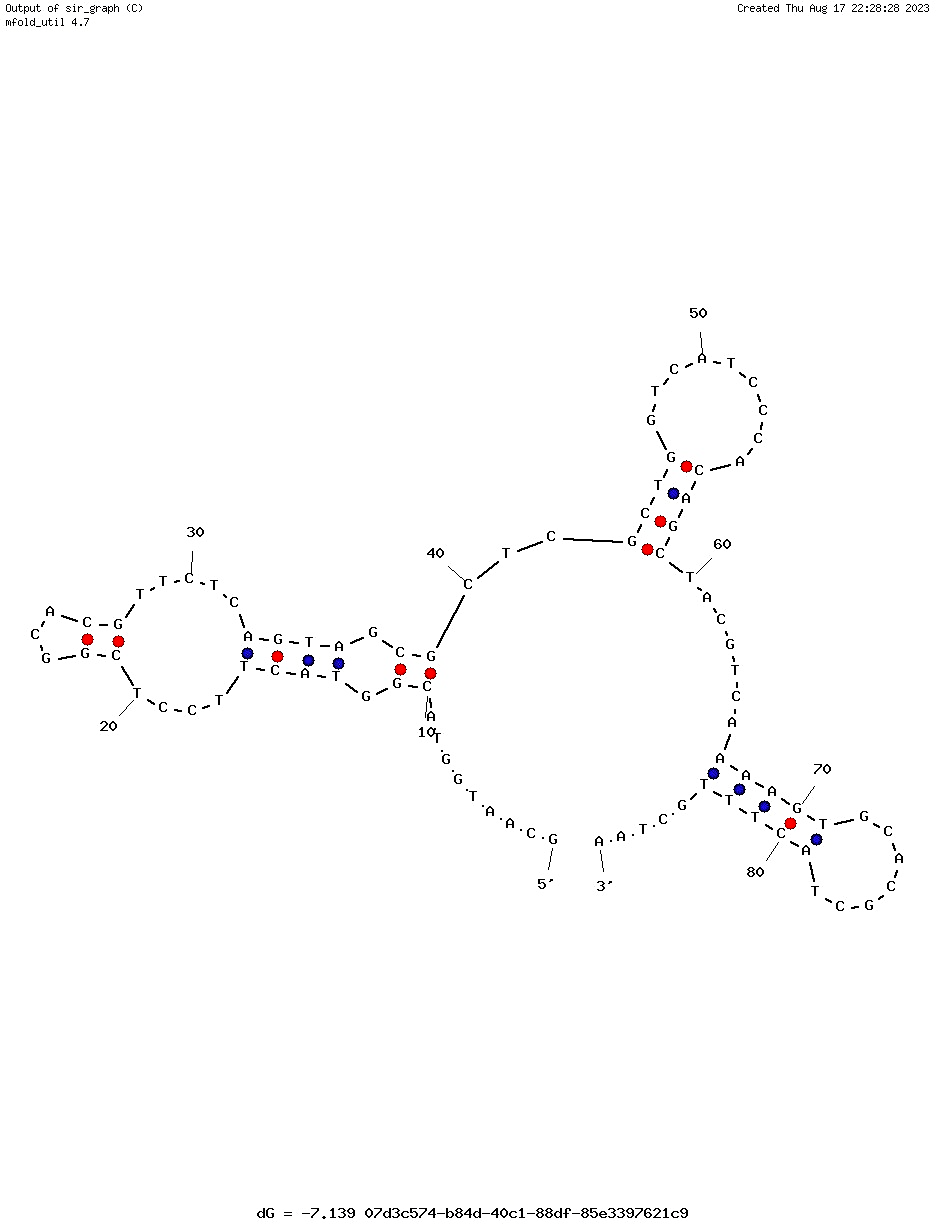


**Fig. S11** Secondary structure of *S. aureus* aptamer at room temperature.

**
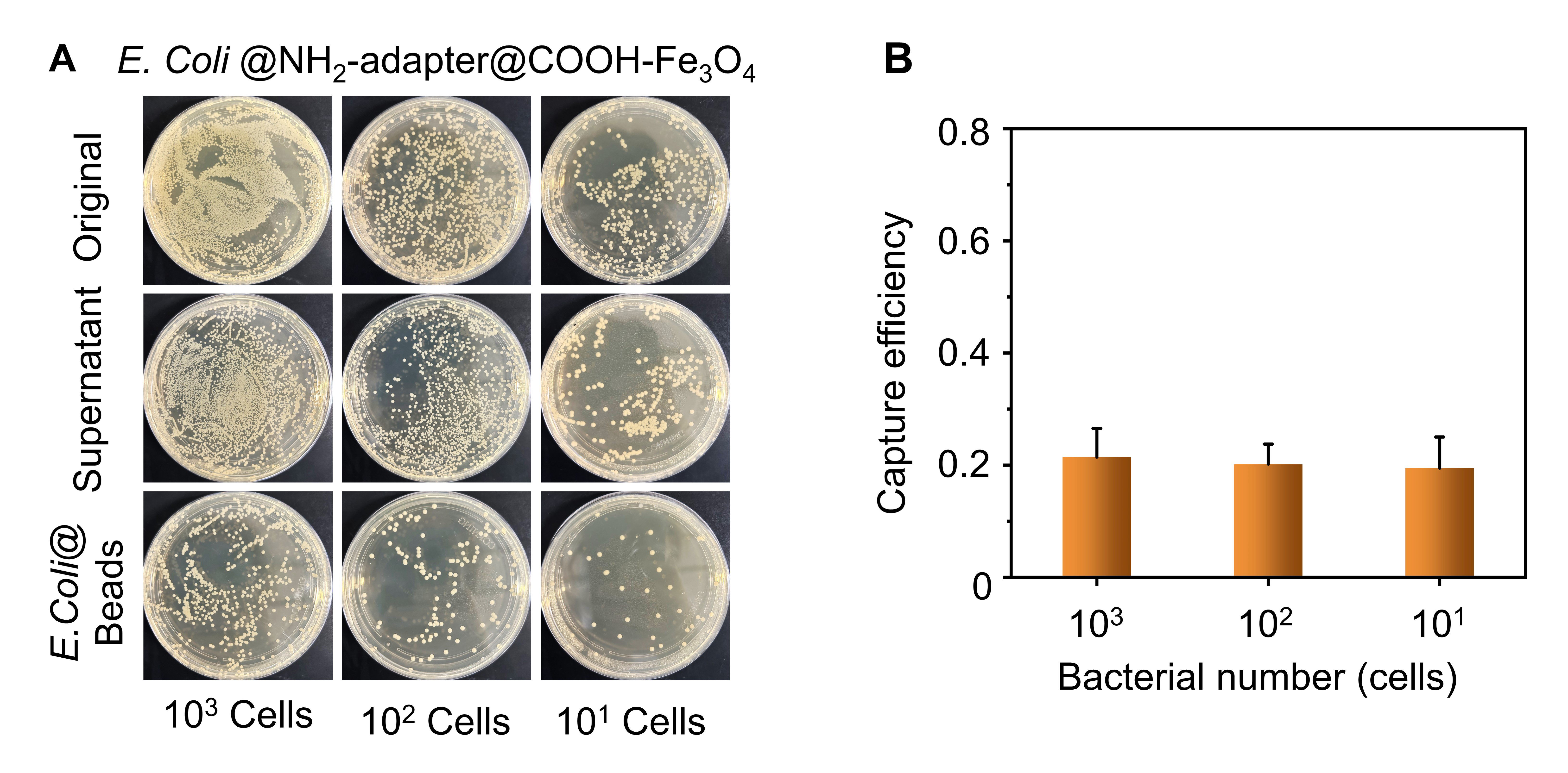
**

**Fig. S12** The agar plate cultivation images (A), and the efficiency of capturing *E. coli* (B), when carboxyl-functionalized magnetic beads were covalently modified with amino-functionalized aptamers.


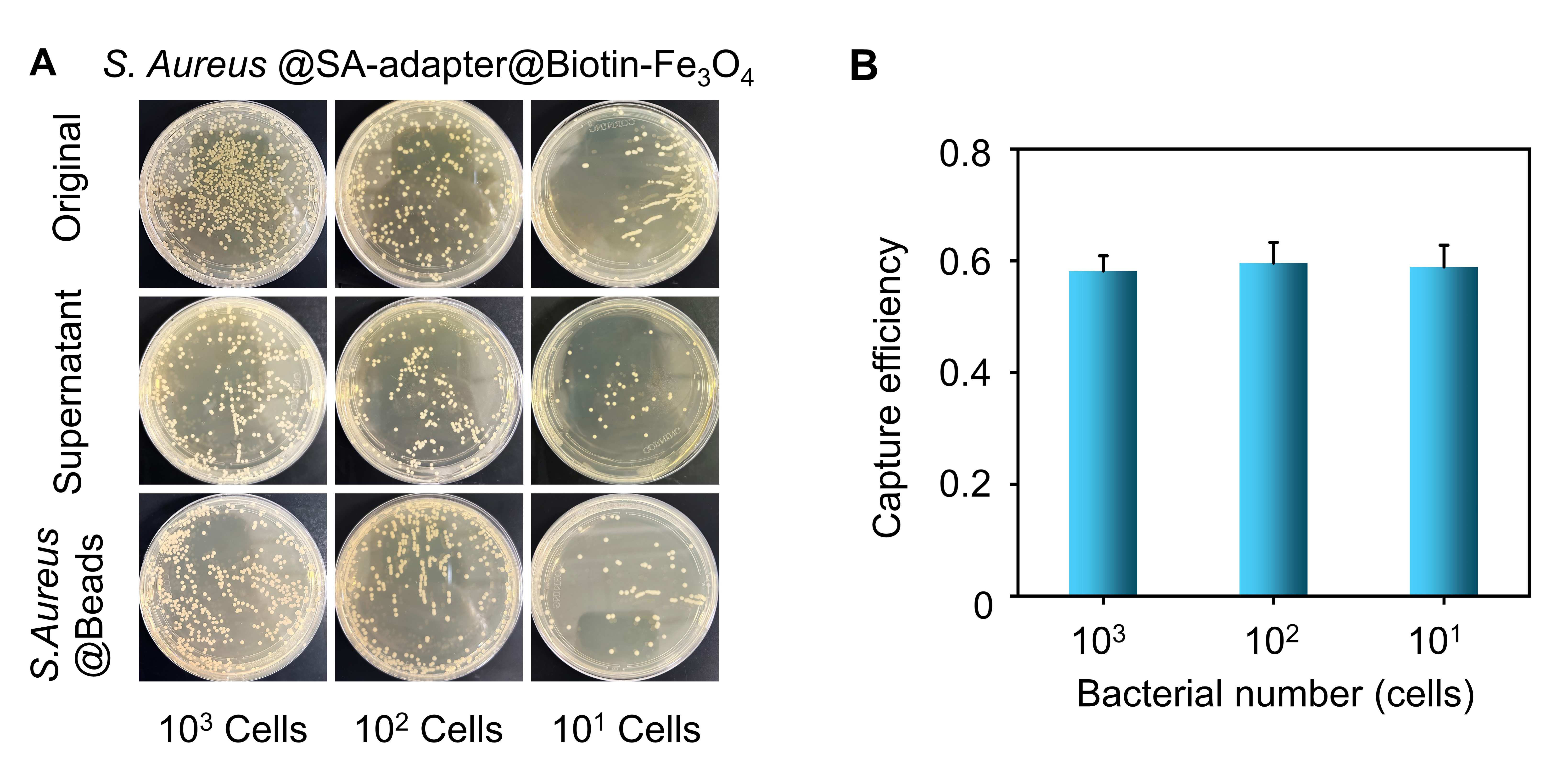


**Fig. S13** The agar plate cultivation images (A), and the efficiency of capturing *E. coli* (B), when the surface of streptavidin-affinity-functionalized magnetic beads were modified with *S. aureus*-specific aptamers.


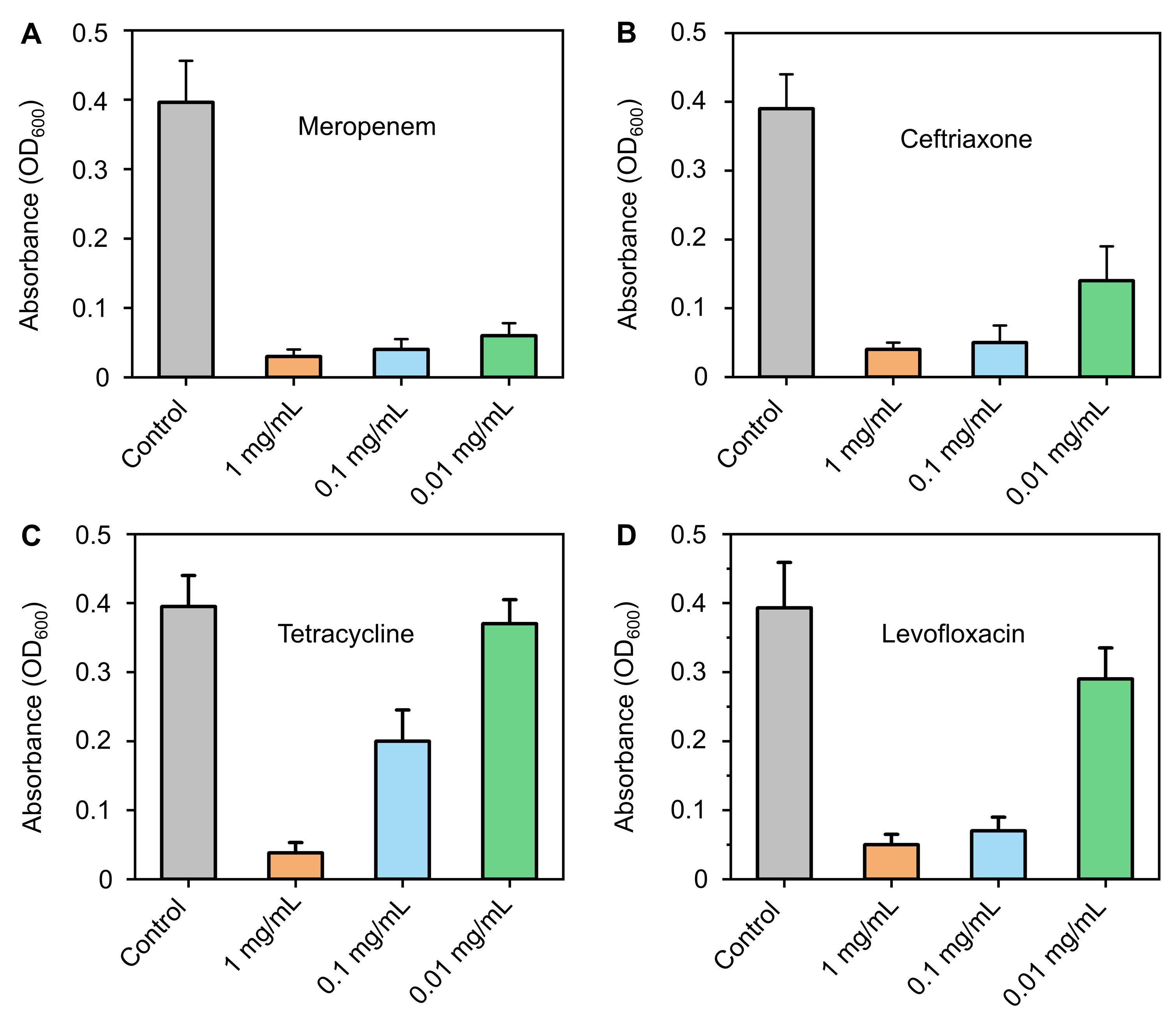


**Fig. S14** The AST responses of *E. coli* to different antibiotics by the bacterial culture method.


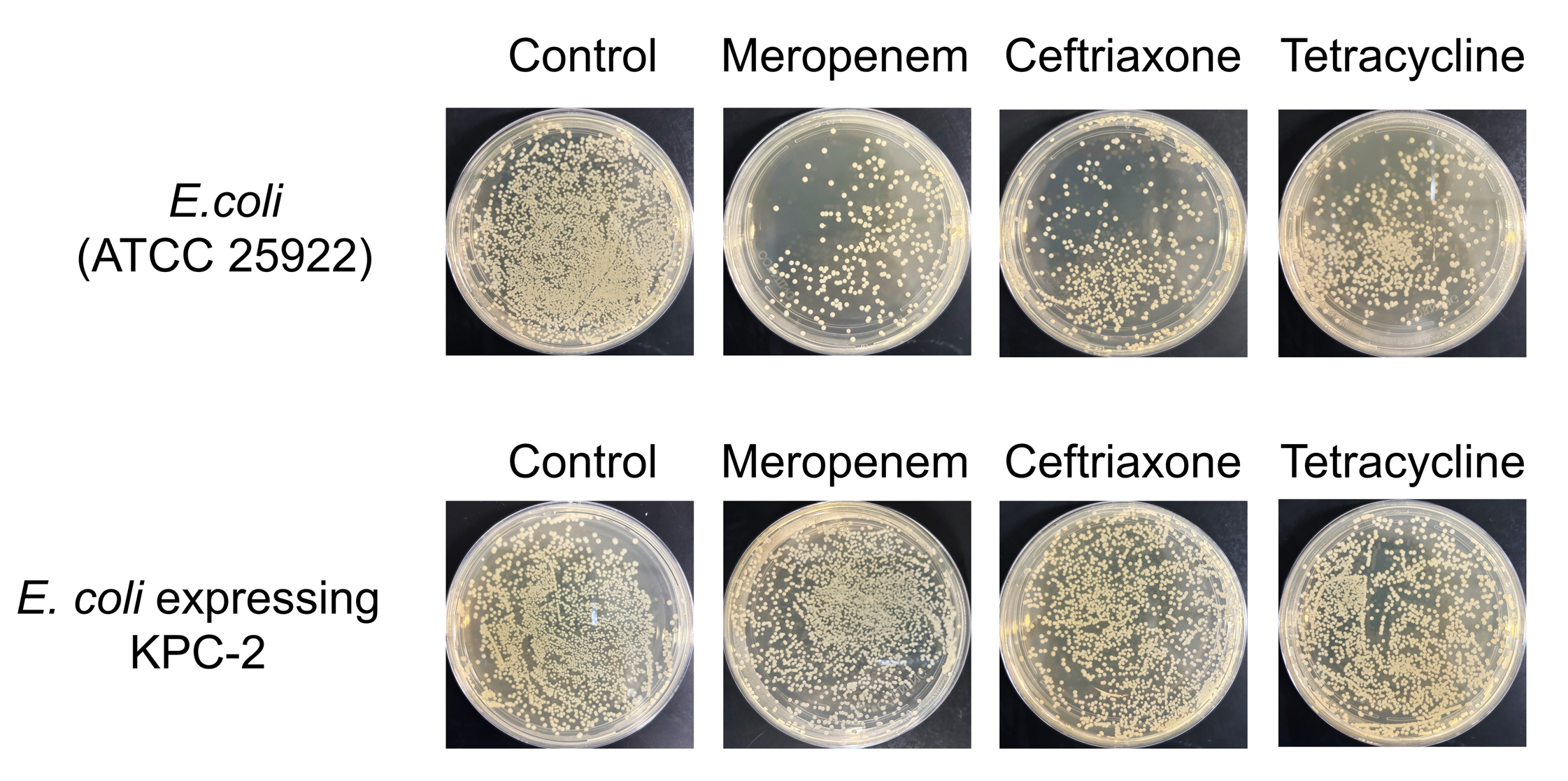


**Fig. S15** The antimicrobial activity of meropenem, ceftriaxone, and tetracycline against *E. coli* (ATCC 25922) and *E. coli* expressing KPC-2 was assessed using the bacterial culture method.

1. **Tables**

**Table S1** The advantages, disadvantages and applications of each method [3-6].

| **Method** | **Advantages** | **Disadvantages** | **Applications** |
| --- | --- | --- | --- |
| **AuNRs-BICC Assay** | **High Sensitivity**: Gold nanorods enhance scattering signals for better detection. | **Costly**: Gold nanorod synthesis can be expensive. | Rapid bacterial detection in environmental monitoring, clinical diagnostics, and food safety testing. |
|  | **Rapid Detection**: Provides quicker results than culture methods. | **Complexity**: Requires dark-field microscopy and advanced imaging equipment. | Suitable for point-of-care testing. |
|  | **Non-invasive**: Direct detection in complex samples without the need for sample extraction/amplification. | **Limited Availability**: Imaging equipment may not be accessible in all settings. |  |
| **Polymerase Chain Reaction (PCR)** | **High Specificity**: Allows for detection of specific bacterial DNA. | **Time-Consuming**: Results take hours to a day, including sample preparation. | High-precision bacterial detection, particularly in clinical and research labs. |
|  | **High Sensitivity**: Can detect low bacterial concentrations. | **Expensive**: Requires sophisticated equipment and reagents. | Used in genetic testing, research, and confirming bacterial species. |
|  |  | **Invasive**: Requires DNA extraction from the sample. |  |
| **Enzyme-Linked Immunosorbent Assay (ELISA)** | **High Throughput**: Allows for testing of multiple samples at once. | **Limited Sensitivity**: Less sensitive for low bacterial concentrations compared to AuNRs-BICC. | Screening of bacterial pathogens in clinical diagnostics and food safety. |
|  | **Relatively Low Cost**: Less expensive than PCR and AuNRs-BICC assays. | **Less Specific**: It detects antigens or antibodies rather than the bacteria directly. | Common in large-scale diagnostics. |
| **Bacterial Culture** | **Gold Standard**: The most reliable method for identifying bacterial species. | **Slow Results**: Can take days for bacterial growth and analysis. | Comprehensive bacterial identification in clinical and research laboratories. |
|  | **Comprehensive**: Allows for bacterial identification, resistance profiling, and other tests. | **Labor-Intensive**: Requires careful handling, incubation, and monitoring of cultures. | Used for detailed analysis of bacterial behavior, resistance, and taxonomy. |
|  |  | **Requires Lab Facilities**: Needs specialized media, equipment, and controlled conditions. |  |
| **MALDI-TOF Mass Spectrometry** | **Efficient:** Extremely rapid identification of bacterial species, high specificity, and ability to analyze multiple samples simultaneously. | **Costly:** High initial cost of equipment, requires cultured bacteria for reliable identification, and involves complex sample preparation for some types of bacteria. | Rapid species identification in clinical and laboratory settings; less ideal for point-of-care testing due to equipment needs. |

**Table S2** The sequences of oligonucleotides.

| **Oligonucleotides** | **Sequence (5’-3’**) |
| --- | --- |
| Alkene-DNA-SH | C_2_H_2_-TTTTTTTGGGGGTTGAGGCTAAGCCGACTCGGAACCCCCA(CH_2_)_6_-SH |
| complementary DNA (cDNA) | GCTTAGCCTCAACCCCCA |
| *E.coli* aptamer-1 | Bio-GCAATGGTACGGTACTTCCCCATGAGTGTTGTGAAATGTTGGGACACTAGGTGGCATAGAGCCGCAAAAGTGCACGCTACTTTGCTAA |
| *E.coli* aptamer-2 | NH_2_-GCAATGGTACGGTACTTCCCCATGAGTGTTGTGAAATGTTGGGACACTAGGTGGCATAGAGCCGCAAAAGTGCACGCTACTTTGCTAA |
| *S. aureus* aptamer | Bio-GCAATGGTACGGTACTTCCTCGGCACGTTCTCAGTAGCGCTC GCTGGTCATCCCACAGCTACGTCAAAAGTGCACGCTACTTTG CTAA |

1. **References**

[1] Q. Zhong, X. Qin, C. Yuan, R. Shi, Y. Wang, Colorimetric determination of sarcosine in human urine with enzyme-like reaction mediated Au nanorods etching, Microchem. J. 165 (2021) 106120.

[2] B. Nikoobakht, M.A. El-Sayed, Preparation and growth mechanism of gold nanorods (NRS) using seed-mediated growth method, Chem. Mater. 15 (2003) 1957-1962.

[3] P. Belgrader, W. Benett, D. Hadley, J. Richards, P. Stratton, R. Jr Mariella, F. Milanovich, PCR detection of bacteria in seven min, Science 284 (1999) 449−450.

[4] H.J. Geertsema, A.C. Schulte, L.M. Spenkelink, W.J. McGrath, S.R. Morrone, J. Sohn, W.F. Mangel, A. Robinson, A.M. van Oijen, Single-molecule imaging at high fluorophore concentrations by local activation of dye, Biophys. J. 108 (2015) 949−956.

[5] C. Jiao, W. Duan, X. Wu, Y. Shang, F. Zhang, M. Zhang, X. Chen, J. Zeng, C. Yang, Multifunctional nanoprobe-amplified enzyme-linked immunosorbent assay on capillary: a universal platform for simple, rapid, and ultrasensitive dual-mode pathogen detection, Anal. Chem. 95 (2023) 11316−11325.

[6] X. Lin, M. Zhao, T. Peng, P. Zhang, R. Shen, Y. Jia, Detection and discrimination of pathogenic bacteria with nanomaterials-based optical biosensors: a review, Food Chem. 426 (2023) 136578.
